# Predictive and prognostic biomarkers of bone metastasis in breast cancer: current status and future directions

**DOI:** 10.1186/s13578-023-01171-8

**Published:** 2023-12-01

**Authors:** Shenkangle Wang, Wenxin Wu, Xixi Lin, Kevin Matthew Zhang, QingLiang Wu, Mingpeng Luo, Jichun Zhou

**Affiliations:** 1 Department of Surgical Oncology, Sir Run Run Shaw Hospital, Zhejiang University School of Medicine, Hangzhou, 310016 Zhejiang China; 2 Biomedical Research Center and Key Laboratory of Biotherapy of Zhejiang Province, Hangzhou, China; 3 The First Affiliated Hospital of Zhejiang Chinese Medical University, Hangzhou, 310014 China; 4 Loma Linda University School of Medicine, Loma Linda, CA 92350 USA; 5 Hangzhou Ninth People’s Hospital, Hangzhou, 310014 China

**Keywords:** Breast cancer, Bone metastasis, Biomarkers, Microenvironment, Prediction, Prognosis, Liquid biopsy, Animal models

## Abstract

The most common site of metastasis in breast cancer is the bone, where the balance between osteoclast-mediated bone resorption and osteoblast-mediated bone formation is disrupted. This imbalance causes osteolytic bone metastasis in breast cancer, which leads to bone pain, pathological fractures, spinal cord compression, and other skeletal-related events (SREs). These complications reduce patients' quality of life significantly and have a profound impact on prognosis. In this review, we begin by providing a brief overview of the epidemiology of bone metastasis in breast cancer, including current diagnostic tools, treatment approaches, and existing challenges. Then, we will introduce the pathophysiology of breast cancer bone metastasis (BCBM) and the animal models involved in the study of BCBM. We then come to the focus of this paper: a discussion of several biomarkers that have the potential to provide predictive and prognostic value in the context of BCBM—some of which may be particularly compatible with more comprehensive liquid biopsies. Beyond that, we briefly explore the potential of new technologies such as single-cell sequencing and organoid models, which will improve our understanding of tumor heterogeneity and aid in the development of improved biomarkers. The emerging biomarkers discussed hold promise for future clinical application, aiding in the prevention of BCBM, improving the prognosis of patients, and guiding the implementation of personalized medicine.

## Introduction

Breast cancer has surpassed lung cancer as the most common cancer worldwide, with an estimated 2.3 million new cases (11.7%) in 2020. It is also the fifth leading cause of cancer-related deaths globally, with 685,000 deaths (6.9%) in 2020 [1]. Bone is the most common site of metastasis in breast cancer, with approximately 70% of women who die from breast cancer experiencing bone metastasis [2]. According to a report by Leone et al., among 9143 stage IV breast cancer patients, the incidence of bone metastasis at the time of initial diagnosis was 37.5%, while visceral metastasis and metastasis to other sites were 21% and 11.9%, respectively [3].

Bone metastasis in breast cancer is challenging to treat and can lead to complications including bone pain, pathological fractures, hypercalcemia, and spinal cord compression, which are collectively known as skeletal-related events (SREs). SREs significantly impact patients' quality of life and reduce survival rates [4, 5]. It is worth noting that breast cancer metastasis can have a long dormant period, and some patients may experience recurrence and metastasis, especially bone metastasis, up to 20 years after the diagnosis of the primary tumor [6]. Considering the profound consequences of bone metastasis and the potential for late recurrence, early detection of bone metastasis and identification of patients at elevated risk of bone metastasis are of utmost importance. Improved screening with biomarkers creates opportunities to provide personalized strategies for early prevention, diagnosis, and treatment.

Here, we summarize the epidemiology of BCBM and the current approaches to diagnosis and treatment. We will also discuss the existing views on the underlying mechanisms of BCBM and the relevant research models. But our primary focus will be on summarizing the studies aimed at discovering potential biomarkers for BCBM.

## The current diagnostic and treatment process for BCBM

There is a considerable body of research on the screening of high-risk populations for BCBM. Studies have identified certain molecular subtypes of breast cancer that are significant risk factors for bone metastasis. Of the main molecular subtypes of breast cancer, Luminal A (LUMA), Luminal B (LUMB1, LUMB2), HER2-positive, basal-like, and triple-negative breast cancer (TNBC), Luminal A and Luminal B subtypes have a significantly higher risk of bone metastasis than the other molecular subtypes [7, 8]. Lymph node involvement and tumor size at the time of breast cancer diagnosis are also associated with an increased risk of bone metastasis [9, 10]. However, these risk factors remain insufficient as clinical and pathological indicators that can effectively predict BCBM.

Diagnostic guidelines for BCBM recommend timely imaging evaluation and biopsy in cases of suspected bone metastasis based on symptoms such as bone pain, pathological fractures, elevated alkaline phosphatase levels, or hypercalcemia [11]. Commonly used imaging modalities include whole-body bone scintigraphy (Emission computed tomography, ECT), X-rays, computed tomography (CT), magnetic resonance imaging (MRI), and positron emission tomography-computed tomography (PET-CT). ECT is the most commonly used method for bone metastasis screening despite having a lower sensitivity and specificity than the more suitable PET-CT [12, 13]. X-rays are the most basic and commonly used method for diagnosing BCBM, but they have lower sensitivity. CT has higher sensitivity and specificity than X-rays and is suitable for detecting metastatic lesions in complex anatomical sites. MRI provides more accurate information about the location, extent, and soft tissue involvement of lesions. Histopathological examination of bone lesions in breast cancer patients is considered the gold standard for diagnosing BCBM (Table 1) [14].

**Table 1:** Comparison of advantages and disadvantages of various imaging modalities for the detection of BCBM

Imaging modality	Pros	Cons
Bone ECT	Provide excellent overall assessment of the skeleton and is recommended as the preferred imaging modality for asymptomatic patients	Unable to differentiate between osteolytic and osteoblastic lesions, nor can it provide a clear indication of the extent of bone destruction
PET-CT	Relatively higher sensitivity or specificity and offers both functional and anatomical imaging capabilities	High cost
X-ray	Basic and commonly used imaging modality; relatively inexpensive and readily available. X-ray can accurately determine the specific location of the lesions and their relationship with the surrounding bones and joints	Low sensitivity
CT	Suitable for complex anatomical structures and can display the size, location, and extent of bone metastases, as well as the bone destruction, repair, and calcification after treatment. It is the preferred modality for evaluating treatment efficacy	It is difficult to distinguish between focal osteoporosis and small disseminated tumors, and it has slightly lower sensitivity
MRI	Excellent soft tissue contrast resolution and can better visualize bone marrow and adjacent soft tissues. It is radiation-free	Bone cortex appears as a dark signal in both T1 and T2-weighted sequences, making it unable to accurately reflect bone repair or damage

**Fig. 1:**
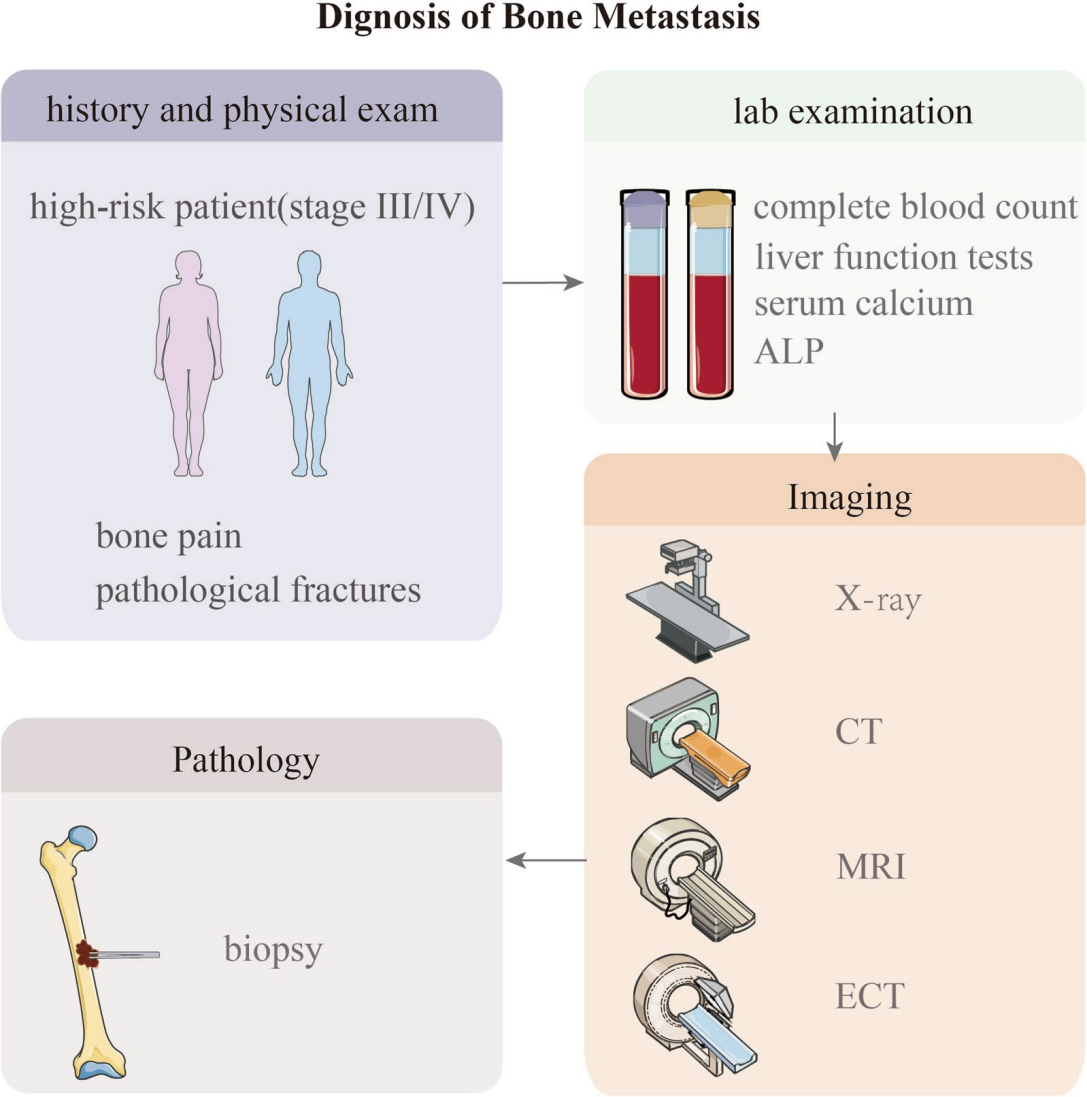
The diagnostic process for bone metastasis in breast cancer

Considering the specificity, sensitivity, cost-effectiveness, and accessibility of various diagnostic methods, imaging evaluation for bone metastasis screening is recommended for patients with stage 3 or higher breast cancer (except for T3N1M0 patients) and those experiencing bone pain, pathological fractures, hypercalcemia, and spinal cord compression or other SREs. NCCN guidelines further recommended tumor markers in the guidelines include alkaline phosphatase, blood calcium, CA15-3, and CA27.29 (Fig. 1). That said, the guidelines also state that BCBM is difficult to evaluate using conventional imaging and emphasize that the recommended tumor markers are not highly specific; abnormality of a single marker alone is insufficient to confirm disease progression [15]. For occult bone metastasis (or when bone metastasis is in a dormant state), the existing imaging and serum markers have limitations in sensitivity and are insufficient to detect metastasis effectively before it becomes overt bone metastasis.

Detecting bone metastasis early and preventing bone metastasis in high-risk populations, can improve the timeliness of treatment and therefore quality of life and prognosis of breast cancer patients. However, the clinical and pathological indicators, imaging, and serum evaluations mentioned above are still insufficient to guide clinicians in accurately screening high-risk individuals for bone metastasis or effectively diagnosing early-stage BCBM (such as clinically undetectable non-visible bone lesions). Therefore, there is an urgent need in the clinical setting for the development of new biomarkers for BCBM, which would facilitate prevention and prediction by clinicians.

When dealing with BCBM, a condition characterized as an end-stage systemic malignant disease, it is imperative to adopt treatment algorithms that primarily focus on systemic therapy. The fundamental treatment modalities include endocrine therapy, chemotherapy, and anti-HER2 therapy. In contemporary clinical practice, new targeted therapies and immunotherapies are also being integrated. It is crucial to customize these therapeutic approaches to cater to the unique needs of individual patients.

The selection of the most appropriate treatment should take into consideration a multitude of factors, including but not limited to 1) Patients' General Health: The overall health and condition of the patients. 2) Intrinsic Molecular Subtypes: The specific molecular characteristics of the tumor. 3) Prior Therapies and Their Side Effects: The patient's history of previous treatments (disease-free interval) and any associated toxicities. 4) Menopausal Status: Whether the patient is premenopausal or postmenopausal. 5) Bone Pain Control: The requirement for managing bone pain. 6) Socioeconomic and Psychological Factors: The social and psychological aspects impacting the patient's well-being.

**Fig. 2:**
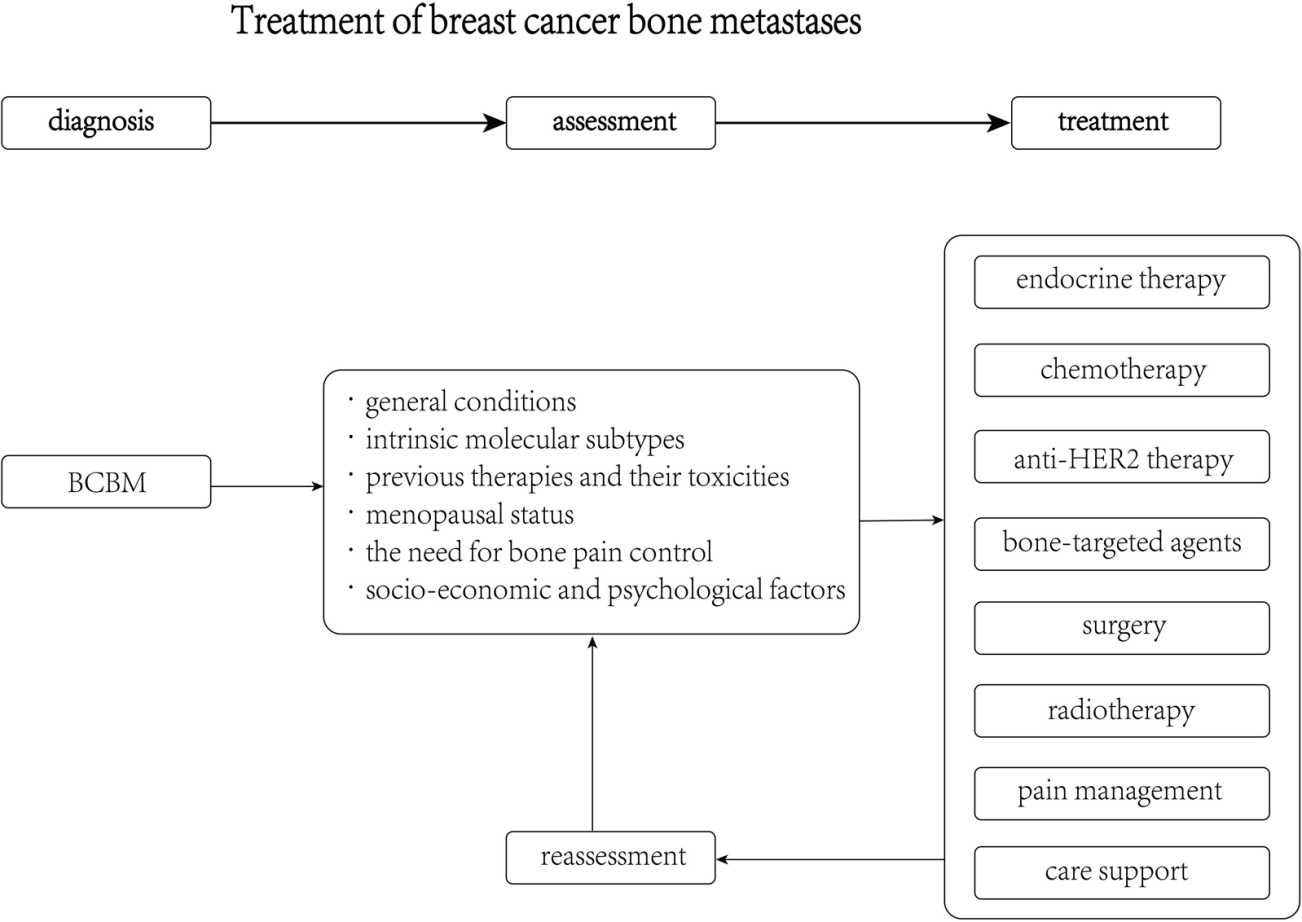
Treatment algorithms of breast cancer with bone metastases

In addition to systemic treatments, the use of bone-targeted agents such as bisphosphonates and denosumab can effectively control the development of skeletal-related events (SREs). For localized management of bone metastases, surgical intervention and radiotherapy are proven and valuable methods. Moreover, it is imperative to provide comprehensive pain management and supportive care to patients. These supportive measures significantly enhance the patients' quality of life (as depicted in Fig. 2). However, it is essential to note that these interventions primarily alleviate symptoms associated with skeletal-related complications and do not offer a direct improvement in patient prognosis [14, 15]. The efficacy and adverse effects of these drugs remain controversial [7–9]. The efficacy and potential adverse effects of these drugs have sparked ongoing debate within the medical community [7–9]. Consequently, there is a pressing demand for more effective treatment strategies that extend beyond the conventional therapies for BCBM. Delving into the molecular mechanisms that underlie BCBM holds great promise in uncovering potential therapeutic targets. In this regard, biomarkers emerge as invaluable tools, offering decision support for the precise selection of treatments tailored to individual patients. Research into the molecular underpinnings of BCBM continually enriches our comprehension of prospective therapeutic targets and clinically significant biomarkers. These insights are pivotal in guiding the choice of suitable treatments for patients.

## Pathophysiology of bone metastasis in breast cancer

Improvements in breast cancer detection and treatment are informed by a sophisticated understanding of BCBM pathophysiology. Bone metastasis in breast cancer is a complex process involving multiple steps and molecular events. Overall, it can be divided into several stages: changes in the pre-metastatic bone microenvironment, rise of osteotropic breast cancer cells, dormancy of cancer cells in the perivascular microenvironment, and the establishment of a vicious cycle [16].

Research suggests that before bone metastasis occurs, breast cancer cells can influence the bone microenvironment by secreting factors to create favorable conditions for metastasis. Factors derived from breast cancer cells, such as IL-1β, may drive bone metastasis by remodeling the bone microenvironment [17]. Additionally, miRNA expressed by the primary breast cancer lesion can influence the pre-metastatic microenvironment. For example, miRNA-183 expressed by breast cancer cells inhibits heme oxygenase-1, promoting osteoclastogenesis [18, 19]. Exosomal miRNA-21 derived from breast cancer cells directly targets osteoclasts, increasing their differentiation and bone-resorbing ability. These factors play important roles in the formation of the bone microenvironment for osteolytic bone metastasis in breast cancer [20]. The roles of miRNA-105 and miRNA-200 in extracellular vesicles produced by breast cancer in the pre-metastatic microenvironment still require further investigation [21–23].

Our current understanding is that the “seeds” of certain breast cancers, which have inherent characteristics that favor bone metastasis, are provided “fertile soil” for metastasis by a shift from a normal bone microenvironment to a pre-metastatic microenvironment [24]. Cells of luminal-type breast cancer are particularly prone to metastasis [7, 8]. The mechanisms underlying osteotropic preference in metastasis are not fully understood, but some studies suggest that they may be related to the differences in pathways and molecular expression between different types of breast cancer. It has been reported that gene expression signals activated by Src are associated with late-stage bone metastasis in breast cancer, and these genes are collectively known as the Src-responsive signature (SRS). 88.4% of ER+ cancer cells are SRS+, while only 23.0% of ER− tumors are SRS+, suggesting that luminal-type cancer cells may gain a survival advantage in the bone microenvironment [25]. Additionally, Smid et al. found that compared to luminal B-type tumors, basal-type breast tumors exhibit more active Wnt/β-catenin signaling, and the loss of Wnt/β-catenin signaling favors bone metastasis in luminal B-type tumors. Inhibition of the classical Wnt signaling pathway in osteoblasts using Dickkopf-1 (DKK1) has been found to promote BCBM [26]. *N*-acetyltransferase 1 (NAT1) is expressed at higher levels in luminal-type tumors compared to TNBC, and it significantly activates the NF-κB signaling pathway, upregulating IL-1B and promoting osteolytic metastasis [27]. A recent study demonstrated that the tumor-secreted factor signal peptide, cubulin domain, epidermal-growth-factor-like protein 2 (SCUBE2), regulated by the ER signaling pathway, mediates bone metastasis in luminal-type breast cancer through immune suppression [28]. These studies suggest that the propensity for bone metastasis in luminal-type breast cancer may stem from multiple molecular mechanisms. In addition to cancer cells themselves, the tumor stromal environment also plays a role in the selection of cancer cells. Zhang et al. found that stromal stem cells in breast cancer secrete chemokine C-X-C motif ligand 12 (CXCL12) and type 1 insulin-like growth factor (IGF1), selecting cancer cells with a tendency for bone metastasis in TNBC. These cancer cells express C-X-C chemokine receptor type 4 (CXCR4), IGF1R, and have high Src activity, making them more likely to metastasize to bone [29]. Bone marrow endothelial cells express CXCL12 and mediate adhesion and recruitment of breast cancer cells that express CXCR4 [30].

**Fig. 3:**
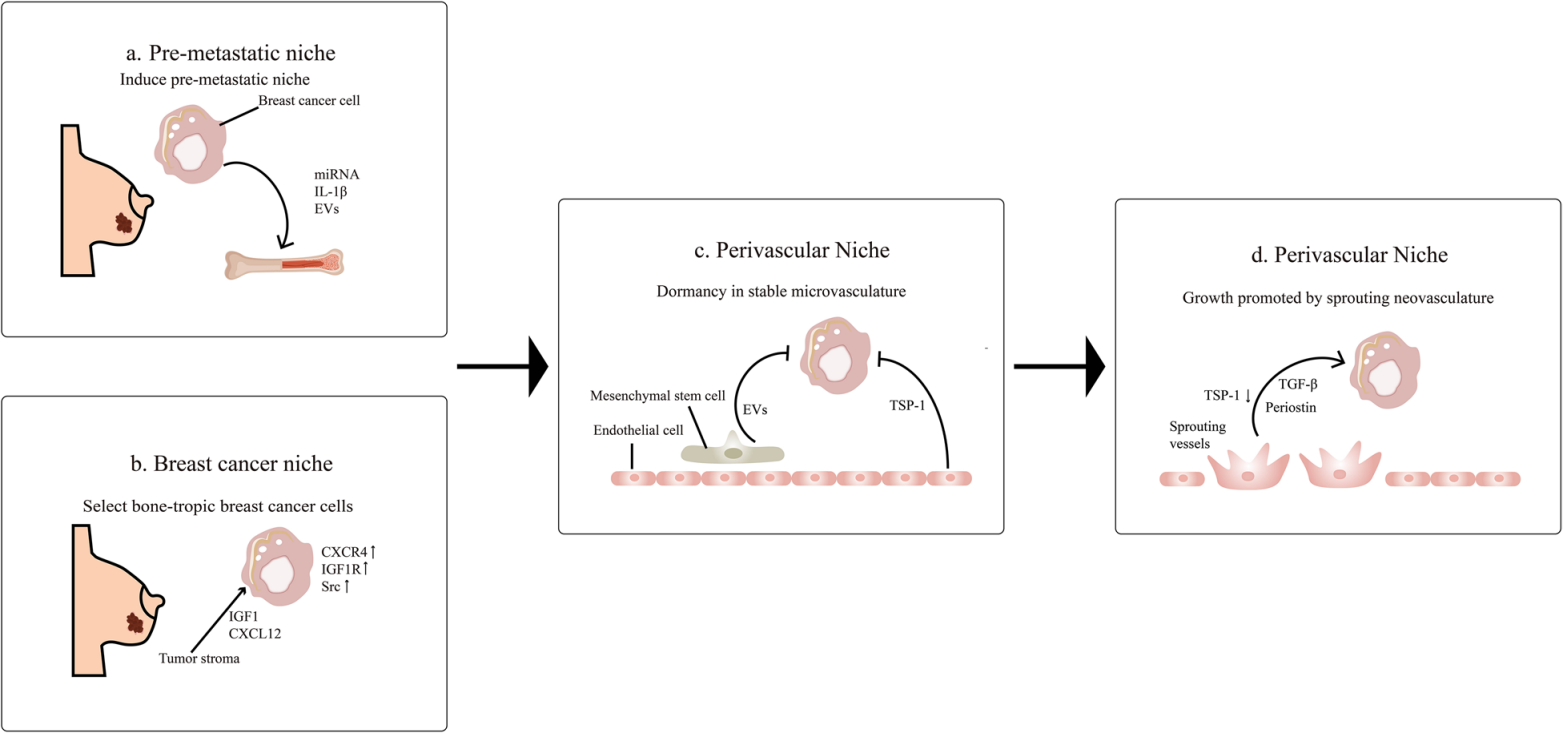
Formation of the pre-metastatic bone microenvironment, rise of osteotropic breast cancer cells, dormancy of cancer cells in the perivascular microenvironment. **a** Breast cancer cells secrete miRNA, IL-1β, and extracellular vesicles to promote the formation of the pre-metastatic microenvironment. **b** The tumor stroma of breast cancer selects bone-metastatic prone cancer cells expressing CXCR4, IGF1R, and high Src activity through CXCL12 and IGF1. **c** The perivascular microenvironment regulates the dormancy of breast cancer cells. Endothelial cells express TSP-1 to promote the dormancy of breast cancer cells. Mesenchymal stem cells deliver extracellular vesicles to induce dormancy in breast cancer cells. **d** Sprouting blood vessels produce periostin, TGF-β, and reduced secretion of TSP-1, stimulating the proliferation of breast cancer cells

After breast cancer cells disseminate into the circulatory system, they may settle with the help of mobilized hematopoietic stem cells (HSCs) in the perivascular microenvironment and enter a dormant state before clinically detectable overt bone metastasis occurs [31]. Endothelial cells expressing thrombospondin-1 (TSP-1) maintain the quiescent state of breast cancer cells [32]. Additionally, mesenchymal stem cells in the bone marrow produce extracellular vesicles that deliver miRNA-222/223 to induce dormancy in breast cancer cells [33, 34]. Clinically, the interval between the detection of primary breast cancer and the appearance of overt bone metastasis can be several years, suggesting that dormancy in breast cancer may persist for a considerable period until cancer cells become active and clinically detectable in the bone metastasis [35]. Many studies have explored the process of reactivation of dormant cancer cells. The remodeling of the perivascular microenvironment is involved in cell activation. Sprouting endothelial cells exhibit reduced expression of TSP-1 and produce periostin and TGF-β1, stimulating tumor cell proliferation [32] (Fig. 3). One hypothesis is that changes in cancer cell metabolism terminate dormancy, but further research is needed to prove this [36]. Understanding the mechanisms of dormancy in breast cancer cells may guide the design of new therapies to prevent the occurrence of overt bone metastasis in breast cancer patients by either enhancing dormancy or eliminating dormant cells. The development of new serological and imaging detection methods aims to detect dormant tumor cells at an earlier stage, which could change the current diagnostic and therapeutic approaches for BCBM, facilitating earlier diagnosis and treatment.

Breast cancer cells interact with the bone microenvironment, resulting in osteolytic lesions, promoting proliferation, and preparing for metastasis to other organs. They regulate the bone microenvironment through multiple mechanisms. Breast cancer cells expressing E-cadherin form heterotypic adhesive connections by binding to N-cadherin on osteoblasts. These adhesive connections promote the activation of the Akt/mTOR signaling pathway in cancer cells, stimulating their proliferation and leading to the formation of overt bone metastasis [37]. The role of mTOR in cancer cell proliferation has been extensively described [38]. Additionally, breast cancer cells in the bone microenvironment secrete parathyroid hormone-related protein (PTHrP), which increases bone resorption caused by osteoclasts [39]. PTHrP also participates in the regulation of the RANKL pathway in osteoblasts. PTHrP activates the cAMP/PKA/CREB signaling pathway, and phosphorylated CREB subsequently binds to the distal RANKL enhancer region to promote RANKL expression [40]. RANKL is an important molecule involved in the regulation of bone formation and resorption. Osteoblasts express RANKL, which binds to the receptor RANK on osteoclast precursors, promoting their differentiation into mature osteoclasts and facilitating bone resorption [41]. RANK is also expressed in breast cancer cells, and RANKL promotes the expression of bone metastasis-related genes (such as IL-11, NCF2, PRG-1, MMP-1) in RANK-positive tumor cells [42]. RANKL produced by osteoblasts can attract cancer cells expressing RANK and induce their migration in the bone microenvironment [43]. From a therapeutic perspective, molecular crosstalk between cancer cells and the bone microenvironment may contribute to drug resistance. Chemotherapy induces osteoblasts to express Jagged1, which interacts with cancer cells and activates the Notch signaling pathway, promoting drug resistance [44]. However, these interactions may also yield therapeutic targets. For example, cancer cells acquire calcium ions by connecting to the gaps between osteoblasts, and this gap junction makes them sensitive to arsenic trioxide treatment [45]. Cancer cells secrete macrophage-stimulating protein (MSP), and the MSP/RON signaling promotes osteoclast activation but does not promote differentiation [46].

**Fig. 4:**
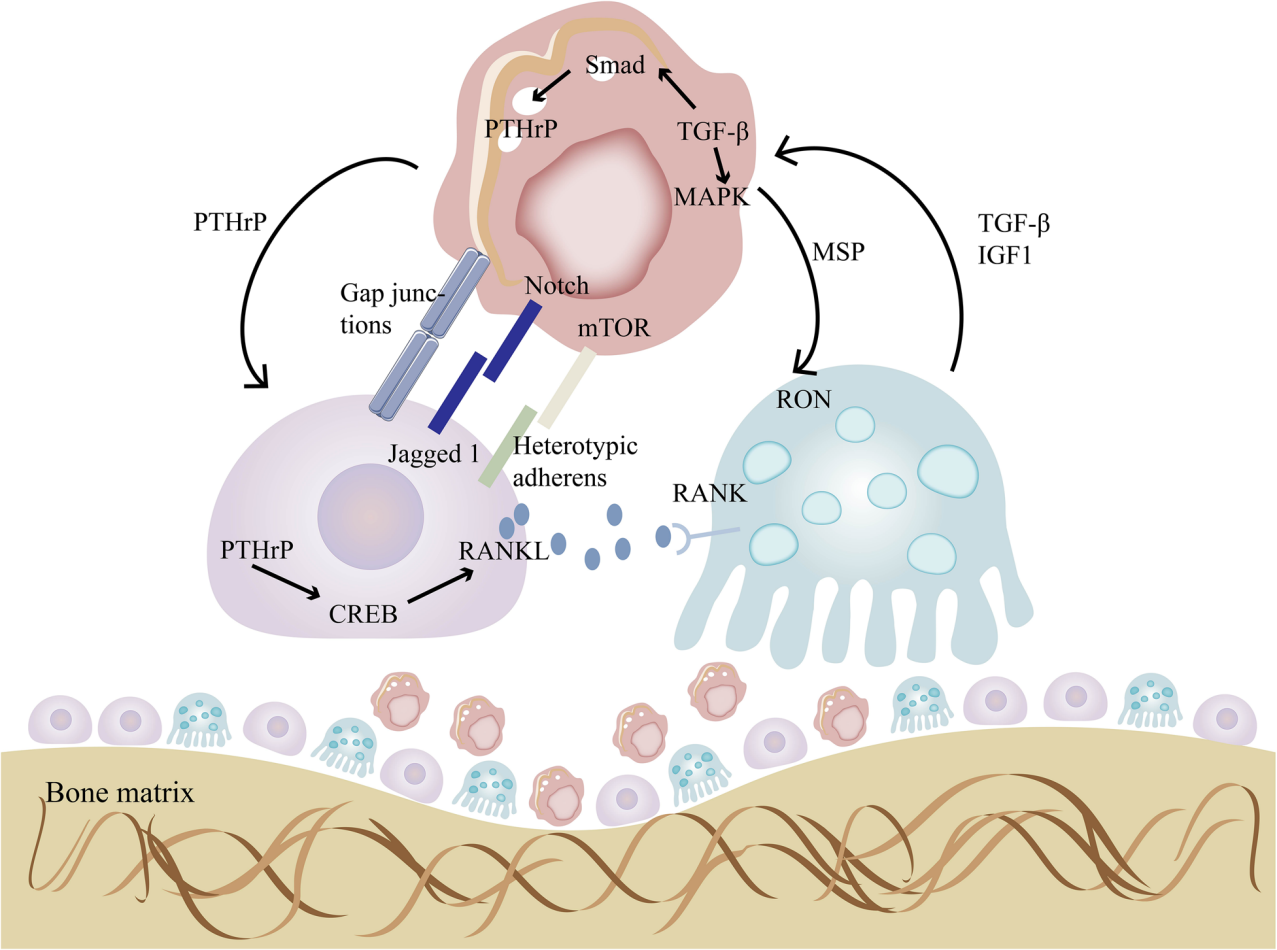
Cell communication between breast cancer cells, osteoblasts and osteoclasts and a vicious circle. Breast cancer cells activate osteoblasts through direct cell–cell interactions and secretion of PTHrP. Osteoblasts, in turn, secrete RANKL to promote osteoclasts differentiation and bone resorption. When bone matrix is resorbed, TGF β, IGF1, and Ca^2+^ are released, promoting the invasion of breast cancer cells

The release of TGF-β, IGF, and Ca^2+^ that comes as a result of cancer-cell promoted bone resorption serves to further improve the survival and proliferation of cancer cells [16]. TGF-β directly acts on breast cancer cells and promotes bone metastasis [47]. In the classical TGF-β signaling pathway, active TGF-β binds to its receptor, TGF-β receptor II (TGF-β RII), which then associates with and activates the TGF-β receptor I (TGF-β RI) on the cell membrane. TGF-β RI phosphorylates downstream signaling molecule Smad3, leading to the activation of PTHrP transcription [48]. Tumor-derived PTHrP acts on osteoblasts, altering the RANKL/Osteoprotegerin (OPG) ratio in the bone matrix, thereby promoting osteoclast maturation and causing metastatic bone destruction [49, 50]. The non-classical TGF-β signaling pathway plays immunosuppressive roles through the activation of p38 MAPK as a Smad-independent pathway [51, 52]. The TGF-β pathway can also promote breast cancer metastasis and invasion through other pathways such as EGFR [53]. TGFβ1 derived from osteoblasts stimulates the AKT/NFκB axis, thereby enhancing the migration of breast cancer cells mediated by transmembrane adhesive receptors integrin β1 and β3 [54]. However, it is worth noting that although TGF-β can promote bone metastasis through Smads, it exhibits anti-cancer effects in pre-cancerous cells, and previous studies have revealed potential mechanisms underlying this paradoxical dual role [55]. In summary, the interaction between breast cancer cells and an altered microenvironment promotes both bone destruction and further proliferation, perpetuating a vicious cycle (Fig. 4). Development of novel targeted drugs to disrupt the interaction between the bone microenvironment and breast cancer cells could pose an effective method for breaking this vicious cycle and controlling the further progression of breast cancer bone metastases.

The events that unfold following breast cancer bone metastasis are of significant concern. As reported by RE Coleman, a study involving 367 female patients with bone metastasis revealed that 228 of them subsequently experienced extra-osseous metastasis [56]. Notably, animal model studies have demonstrated that mice with bone metastasis exhibit a notably higher tumor burden in distant organs. This observation suggests that tumor cells exposed to the bone microenvironment possess significantly enhanced secondary metastatic capabilities [57]. While the specific factors and processes governing metastasis from bone to other organs remain to be fully elucidated, it is evident that the bone microenvironment plays a crucial role in augmenting the metastatic potential of tumor cells. Research has indicated that following the formation of bone metastatic lesions, EZH2 within the metastatic microenvironment enhances invasive capabilities and stem cell-like properties through epigenetic reprogramming. This, in turn, facilitates the further dissemination of cancer cells [57].

Furthermore, recent investigations suggest that immune regulation also plays a role in secondary metastasis. Apoptotic bodies originating from osteoclasts have been found to suppress the function of naive CD8^+^ T cells via Siglec15, thereby promoting multi-organ metastasis in the late stages of breast cancer bone metastasis [58].

## Animal models

Knowledge of the mechanisms of BCBM and the development of useful biomarkers are dependent on research with the appropriate animal models. In vivo models offer greater advantages over in vitro work in demonstrating the interactions between cancer cells and the microenvironment, as well as simulating complex metastatic processes. The ideal animal model would provide a human-like environment for studying the mechanisms of BCBM and help uncover deeper insights into the process [59]. Several animal models for BCBM have been established, each with its own characteristics. We will discuss the advantages and limitations of these models.

Intracardiac injection has been widely used in bone metastasis research [60]. Injection of cancer cells into the left ventricle produces a variety of bone metastases at sites including the tibia, femur, humerus, and spine [61]. This model can also be used to develop osteotropic cell lines [62].

Alternatively, cancer cells can be injected through the iliac artery, which specifically delivers cancer cells to hind limb bones, avoiding the spread to other organs and preventing premature death and allowing the bone metastatic lesions to develop fully [63]. This technique has been useful in studying further dissemination from bone to other organs [57]. The drawback is that procedure requires microscopic surgery, making it challenging to apply this technique in experiments that require a large number of animal subjects.

Researchers have recently developed a caudal artery injection protocol that selectively delivers a large number of cancer cells to the bone marrow of the hind limbs. Compared to iliac artery injection, this method is easier to implement and causes less trauma to the animal [64, 65]. This model has been applied in the study of bone metastasis in breast cancer and prostate cancer [66, 67]. Femoral artery injection is another viable option for delivering cancer cells into hind limb without causing metastases in other sites [68].

Intraosseous injection is the most direct method of confining cancer cells to the bone and reducing the possibility of metastasis to other organs [27, 69]. However, the invasive procedure causes bone injury, and the metabolic changes during the bone repair process may cause unwanted confounding effects [70]. Additionally, this type of model is only capable of demonstrating the last step in the process of metastasis.

The above-mentioned circulation injection models offer unique advantages, but they bypass the earliest stages of metastasis. They cannot be used to study the interaction between tumor cells with bone tropism and the extracellular matrix at the primary site. The orthotopic injection is a more suitable model, in which breast cancer cells are injected into the mammary fat pad of mice, where they develop into primary tumors and subsequently generate bone metastases. This model allows for the complete process of metastasis to occur. The cell lines used in this spontaneous metastasis model are 4T1.2 and 4T1.13, which are sublines of the 4T cell lines and have been shown to have a high propensity to metastasize to lymph nodes, lungs, and bones [71, 72]. Compared to xenograft models that require immunodeficient hosts for cancer cell growth, this model uses immunocompetent mice, allowing for the study of the interaction between cancer cells and the host immune system during bone metastasis [73].

**Fig. 5:**
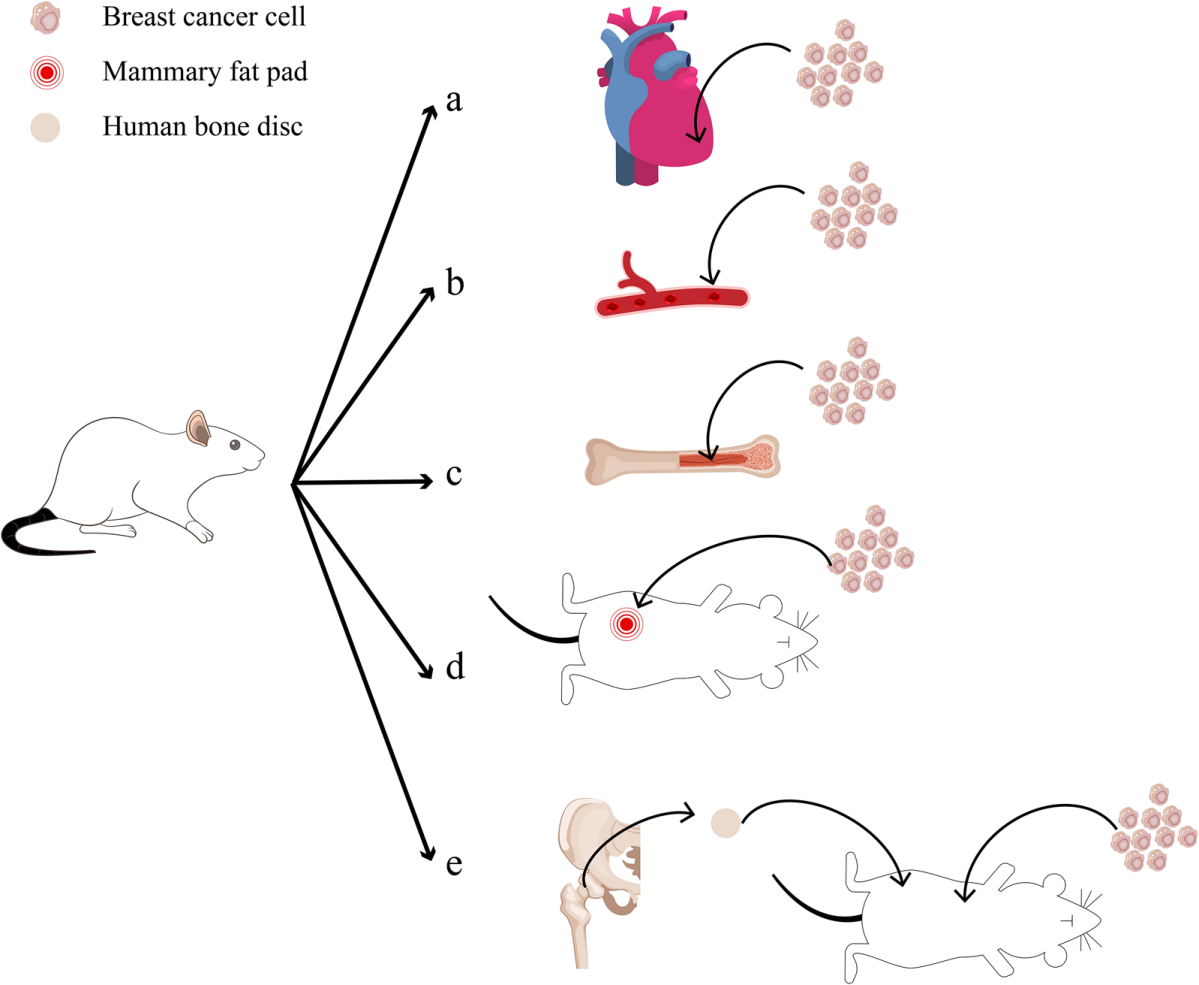
Animal models of BCBM. **a**, **b** Intracardiac/intraarterial injection models: left ventricle injection, iliac artery injection, caudal artery injection, femoral artery injection. **c** Intraosseous injection models: tibia bones injection, femur bones injection. **d** Orthotopic injection model: mammary fat pad injectione. **e** Humanized model: implantation of human bone chips into mice

Though the aforementioned models have significantly advanced knowledge of BCBM, researchers aim to establish models that better reflect the conditions in human body. Humanized models provide tumor cells with microenvironments more similar to the human body, allowing for a closer approximation of tumor progression in patients. Some humanized models are relevant to the study of the interaction between cancer cells and the human bone microenvironment. A recently invented model involves implanting tissue from the femoral heads of patients undergoing hip replacement surgery under the skin of immunodeficient mice with subsequent orthotopic injection of cancer cells. The graft exhibits metabolic activity, bone remodeling and can undergo bone metastasis. This model demonstrates spontaneous metastasis of injected breast cancer to the transplanted bone and may be used for studying the driving factors of metastasis and the bone microenvironment of metastasis research [74] (Table 2 and Fig. 5).

**Table 2:** Animal models of BCBM

Type of model	Cell lines	Animal	Methodology (Cell injection site)	Ref
Intracardiac/intraarterial injection model	MDA-MB-231	BALB/c nu/nu mice	Left ventricle	[61]
	MCF7, 4T1, 4T07, MDA-MB-361, MDA-MB-231, MDA-MB-436	4–6 weeks old mice	Iliac artery	[63]
	MDAMB-231, MCF7, 4T1, E0771	NOD-SCID, SCID, BALB/c, C57B/ 6 albino	Caudal artery	[64]
	AT-3	C57BL/6J mice	Femoral artery	[68]
Intraosseous injection model	T47D	BALB/c mice	Tibia bones	[27]
	MDA-MB-231	BALB/c nu/nu mice	Femur bones	[69]
Orthotopic injection model	4T1	BALB/c mice	Mammary fat pad	[71]
Humanized model	MDA-MB-231, MCF7, T47D, Patient-derived xenograft (PDX) tumor cell	NOD/SCID NOD/SCIDγ mice	Implantation femoral head tissues derived from patients undergoing hip replacement surgery into the left and right sides of the mouse body, followed by intracardiac or orthotopic injection of tumor cells	[74]

In summary, the continuous development of BCBM models provides the tools needed to further elucidate the mechanisms of BCBM while moving the world of bench research ever closer to the goal of clinical utility. It aids in the exploration of new predictive and prognostic biomarkers and provides a reliable platform for the development of potential therapeutic targets.

## Biomarkers for BCBM

Using appropriate models and research techniques, researchers have explored potential biomarkers for BCBM, including various proteins, genes, and circulating cells. These biomarkers can be broadly classified into two categories: prognostic biomarkers and predictive biomarkers. Prognostic biomarkers provide information about recurrence and may be associated with progression-free survival (PFS) in metastatic disease patients. Predictive biomarkers can predict a patient's response to treatment or be used for monitoring during treatment to determine if the patient is benefiting from the therapy [75].

### Prognostic biomarkers

Prognostic biomarkers provide information about recurrence and may be associated with progression-free survival in metastatic disease patients [75].

#### Proteins

Several studies have investigated the value of protein molecules in predicting the occurrence of bone metastasis.

Nuclear p21-activated kinase 4 (nPAK4) can promote BCBM by inhibiting leukemia inhibitory factor receptor (LIFR). LIFR plays an important role in BCBM. It activates STAT3 to promote the dormancy of breast cancer cells and triggers the Hippo kinase cascade, leading to the functional inactivation of yes-associated protein (YAP), thus inhibiting bone metastasis [76, 77]. PAK4 directly regulates LIFR in a 17β-estradiol (E2)-dependent manner. Under E2 stimulation, PAK4 forms a complex with ERα, which is recruited to the target gene LIFR of ERα, downregulating LIFR expression and promoting the metastasis and invasion of breast cancer cells. PAK4 can also inhibit E-cadherin and promote epithelial-mesenchymal transition (EMT) [78]. Li et al. found that nPAK4 expression significantly leads to inhibition of LIFR and is associated with ERα+ BCBM. The prognostic value of nPAK4 was confirmed in clinical samples. In 187 cases of non-bone metastatic breast cancer (NMBC) and 95 cases of bone metastatic breast cancer (BMBC) specimens, the nPAK4 score in the BMBC group was significantly higher than that in the NMBC group. Moreover, in ERα+ BCBM patients, the level of nPAK4 was associated with shorter bone metastasis-free survival [78].

Macrophage-capping protein (CAPG) and PDZ domain–containing protein GIPC1 (GIPC1) are also involved in BCBM. CAPG, an actin-binding protein, may enhance tumor invasion by altering cytoskeletal dynamics [79]. It has been shown to interact with protein arginase methyltransferase 5 (PRMT5), leading to enhanced transcription of stanniocalcin-l (STC-1), a factor that can activate PI3K/AKT pathway and thereby promote breast cancer cell invasion [80, 81]. GIPC1 silencing can result in cell cycle arrest in breast cancer cells and regulate cell adhesion and motility through interaction with syndecan-4 (SDC4) via the PI3K signaling pathway [82]. Using quantitative proteomics, Westbrook et al. discovered that the expression of CAPG and GIPC1 in the bone metastatic variant of human breast cancer cell line MDA-MB-231 was higher than in the parental non-bone metastatic cells. After preliminary biomarker screening, they validated the findings using immunohistochemical staining on tumor tissue microarrays of AZURE trial patients. They further explored the relationship between protein expression, clinical variables, and distant metastasis using COX regression analysis. The study included 427 patients in the training set and 297 patients in the independent validation set. The results revealed that patients with high expression of both CAPG and GIPC1 in the primary tumor were more likely to experience first distant recurrence is skeletal and had a higher probability of death. One advantage of CAPG and GIPC1 as prognostic biomarkers is their specificity, as the researchers did not find any association between the composite biomarkers and non-bone metastasis development [83].

Using similar approach, Westbrook et al. discovered that the expression level of dedicator of cytokinesis protein 4 (DOCK4) was higher in the bone metastatic variant of human breast cancer cell line MDA-MB-231 compared to the parental non-bone metastatic cells. They further validated this finding through western blotting, confirming the potential of DOCK4 as a biomarker. In clinical validation, the correlation between high DOCK4 expression and invasive tumors was confirmed in a tissue microarray containing 345 samples. Subsequent research using samples from 689 patients in the AZURE trial found that adjusted Cox regression analysis showed that high DOCK4 expression was associated with a higher risk of bone metastasis occurrence [84]. DOCK4 mediates the migration and invasion of MDA-MB-231 cells. DOCK4 forms a complex with engulfment and cell motility (ELMO), and under the activation of RhoG, the ELMO-DOCK4 complex translocates from the cytoplasm to the plasma membrane, activating Rac to promote lamellipodia formation and cell migration [85].

RANKL is a factor regulating bone remodeling [86]. It also enhances the invasive ability of breast cancer, inducing spontaneous formation and early metastasis [87]. Studies have found that high serum levels of RANKL have been associated with an increased risk of bone metastasis in breast cancer patients. In a retrospective analysis, Rachner et al. studied the relationship between serum RANKL levels and bone metastasis and survival in a cohort of 509 patients with primary breast cancer. During the follow-up period, out of 413 patients available for evaluating distant metastasis, 23 developed bone metastasis. According to serum RANKL levels, patients were divided into high and low groups based on the median. The high RANKL group had an 87.5% increased risk of bone metastasis compared to the low group. Patients with the highest quartile of serum RANKL concentration had a fivefold increased risk of bone metastasis compared to those with the lowest quartile. Additionally, patients who experienced bone metastasis showed significantly elevated levels of RANKL/OPG [88].

Another bone remodeling regulator is prolactin (PRL). It promotes the secretion of osteoclastogenic factor sonic hedgehog (SHH) by breast cancer cells, thereby activating the Hedgehog pathway in osteoclasts and promoting bone resorption [89]. The HH pathway upregulates PTHrP and regulates the RANKL pathway, both of which are involved in the process of BCBM [90]. Another study has found that PRL can also activate Nek3 to regulate Rac1, increasing the invasiveness of breast cancer cells [91]. In a study conducted by Ashley Sutherland, the researchers investigated the levels of prolactin receptor (PRLR) using quantitative immunohistochemistry in 134 primary breast cancer samples and matched samples of 17 primary breast cancer and bone metastasis. They aimed to explore the relationship between PRLR expression in primary breast tumors and the time of bone metastasis occurrence. The Cox proportional hazards regression model demonstrated that high expression of PRLR was associated with an earlier occurrence of bone metastasis [89].

IL-1B also plays an important role in bone metastasis. It promotes EMT in breast cancer cells and regulates osteolysis, promoting cancer cell proliferation [92, 93]. IL-1B also contributes to reorganization of the actin cytoskeleton of MCF-7 cells through the PI3K/Rac axis, enhancing cancer cell invasion [94]. Using immunohistochemical staining, Nutter et al*.* discovered that the expression level of IL-1B protein was increased in bone metastatic cells compared to the primary breast cancer cells. Subsequently, in a tissue microarray containing 150 cases of stage II and III primary breast tumors, IL-1B was measured using immunohistochemistry. The median follow-up period was 84 months. The study found a significant correlation between IL-1B expression and bone metastasis [62].

A recent study found that the specific expression of SCUBE2 in luminal-type breast cancer is associated with shortened bone metastasis-free survival. By analyzing the expression of secreted SCUBE2 protein in the serum of patients from the Qilu cohort, Wu et al. observed an upregulation of SCUBE2 expression in bone metastasis patients (n = 13) compared to patients without distant recurrence (n = 23), confirming the prognostic value of SCUBE2 in luminal-type breast cancer patients. Mechanically, regulated by ER signaling in luminal-type breast cancer, SCUBE2 acts in an autocrine manner on cancer cells, leading to Hedgehog signal activation, osteoblast differentiation, and immune suppression-mediated bone metastasis [28].

Bone turnover markers also provide prognostic information about bone metastasis. They can be divided into bone formation markers and bone resorption markers. Bone metastasis disrupts the balance between bone formation and bone resorption, leading to changes in the levels of these markers [95]. Procollagen type I N-terminal propeptide (P1NP) is a marker of bone formation, while C-telopeptide of type-1 collagen (CTX) and cross-linked carboxyterminal telopeptide of type I collagen (1-CTP) are markers of bone resorption. Brown et al. measured the levels of P1NP, CTX, and 1-CTP in the blood of 872 patients participating in the large randomized AZURE trial to investigate their prognostic and predictive relationship with metastatic events. They found that elevated serum levels of P1NP, CTX, or 1-CTP were associated with an increased risk of bone metastasis, with P1NP being the most sensitive marker [96]. The prognostic value of P1NP was further confirmed in another study conducted by Colomb et al*.* They measured the serum levels of P1NP, CTX, IL-6, and osteocalcin in the blood of 164 pre-treatment stage I-III breast cancer patients to explore the relationship between these markers and bone metastasis and overall survival. They found that in stage I–III breast cancer patients, a serum P1NP level ≥ 75 ng/mL indicated a higher risk of bone metastasis and shorter overall survival [97] (Table 3).

**Table 3:** Prognostic protein biomarkers for BCBM

Biomarker	Increased or decreased	Clinical samples	Potential molecular mechanism	Refs.
PAK4	Increased	187 NMBC patients 95 BMBC patients	nPAK4 inhibits LIFR and promotes osteolytic bone destruction in ERα-positive breast cancer	[78]
CAPG, GIPC1	Increased	724 breast cancer patients from AZURE trial	CAPG promotes cell migration and invasion GIPC1 regulates cell cycle, cell adhesion, and motility	[79, 82, 83]
DOCK4	Increased	689 breast cancer patients from AZURE trial	DOCK4 regulates breast cancer cell migration and metastasis	[84, 85]
RANKL	Increased	413 breast cancer patients	RANKL enhances invasion and metastasis of breast cancer and regulates bone microenvironment	[87, 88]
PRLR	Increased	134 breast cancer patients	PRL promotes the secretion of SHH by breast cancer cells, facilitating osteolysis and increasing invasiveness	[89, 91]
IL-1B	Increased	150 stage II/III primary breast cancer patients	IL-1B promotes EMT in breast cancer cells, enhancing their invasive capacity	[62, 92–94]
SCUBE2	Increased	Blood samples from 23 breast cancer patients without distant recurrence, 13 cases with bone metastasis	SCUBE2 regulates the immunosuppressive osteogenic microenvironment	[28]
CTX, ICTP, P1NP	Increased	872 breast cancer patients from AZURE trial 164 pre-treatment stage I-III breast cancer patients	CTX, ICTP and P1NP are associated with bone remodeling	[96, 97]

**Fig. 6:**
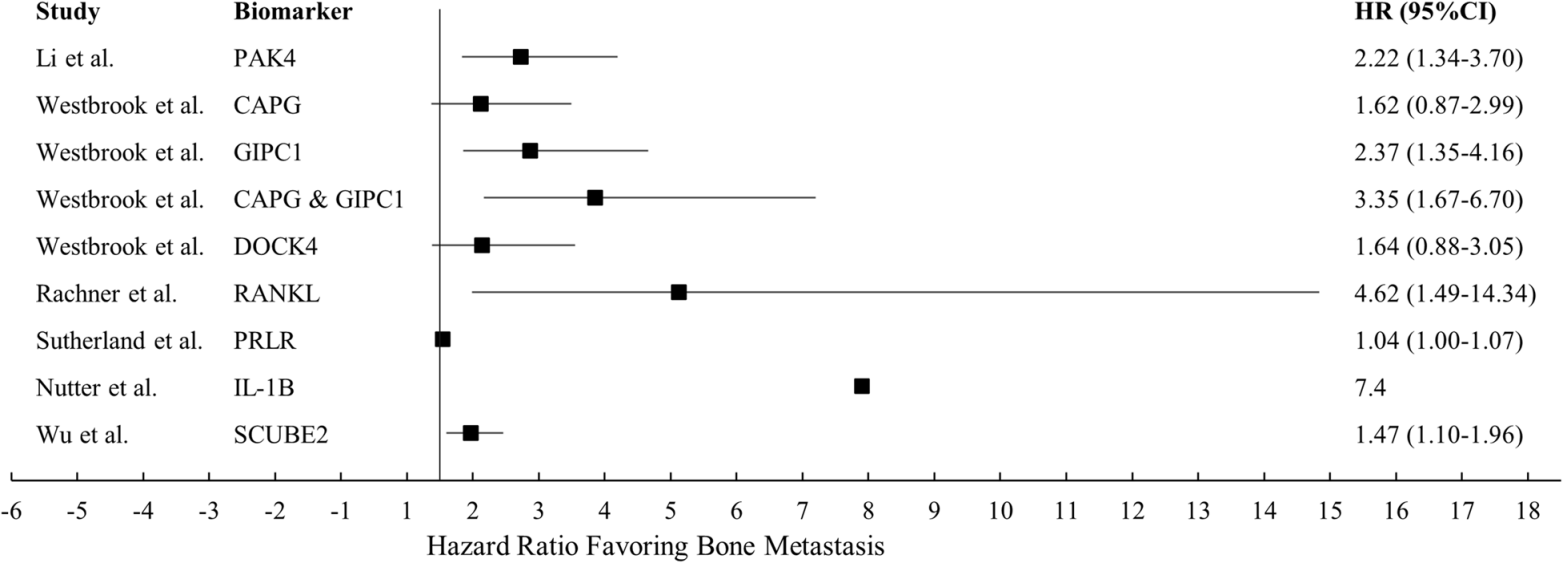
Forest plot of protein biomarker hazard ratios for bone metastasis-free survival

Most, if not all biomarkers that have been published are statistically significant. The confidence of each study also varies greatly. The protein biomarkers mentioned above are presented in a forest plot (Fig. 6). More research and careful analysis in the future will tell us which of these are clinically significant.

#### RNAs and genes

RNAs and genes play a crucial role in BCBM. The advancement of genomics has facilitated a better understanding of the mechanisms underlying BCBM. Several studies have utilized microarray data analysis, qRT-PCR, FISH assay or other approaches to identify RNAs and genes with prognostic implications.

It has been found that many non-coding RNAs associated with bone metastasis have prognostic value. A study reported that breast cancer cells deliver extracellular vesicles containing miRNA-19a to preosteoclast cells, inhibiting PTEN expression. PTEN reduction induces the activation of NFκB and AKT signaling pathways, promoting osteoclast differentiation and bone resorption activity. Uptake of extracellular vesicles containing miRNA-19a by preosteoclast cells is enhanced in a concentration-dependent manner. Integrin binding sialoprotein (IBSP) secreted by ER+ breast cancer cells can recruit preosteoclast cells, creating a microenvironment enriched with preosteoclast cells, which facilitates miRNA-19a regulation of bone resorption [98]. Wu et al. investigated expression of protein secretion and miRNAs contained in EVs in MCF7 bone metastatic cell variants. Although 157 miRNAs were significantly increased in the variant cells, only three miRNAs showed significant elevation in the blood of breast cancer patients (n = 23) compared to healthy individuals (n = 22). By comparing the protein secretion differences between bone metastatic cell variants and their parental cells, and subsequently detecting these proteins in the biopsies from BCBM patients, the researchers identified IBSP as a potential biomarker. To further elucidate the relationship between these potential biomarkers and bone metastasis in ER+ breast cancer, a Kaplan–Meier analysis was performed on 278 breast cancer patients. Survival analysis indicated that extracellular vesicle miRNA-19a and IBSP only had suggestive roles in predicting bone metastasis risk in ER+ patients. Among 41 cases of ER+ breast cancer patients with metastasis, patients with elevated levels of both IBSP and miRNA-19a showed earlier metastasis within 1000 days [98].

A study conducted by Adam Artigues reported the dysregulation of miRNA-30b-5p in breast cancer metastasis patients. This is another biomarker that can be detected in blood. The researchers first measured the levels of miRNA-30b-5p in tissue samples and then confirmed its potential as a biomarker in liquid biopsy using peripheral blood samples. They found that miRNA-30b-5p was a valuable non-invasive diagnostic biomarker. The expression level of miRNA-30b-5p in bone metastases was significantly higher than in other metastatic sites. However, it should be noted that although miRNA-30b-5p was found to have predictive value for distant metastasis, with 63 bone metastasis patients included in the cohort, 30 of them had metastasis in other sites. Therefore, further research is needed to determine whether miRNA-30b-5p can specifically predict bone metastasis [99].

miRNA-16 and miRNA-378 have the potential to be used in liquid biopsy as well. Mechanically, miRNA-16 may enhance osteoclast function and bone destruction caused by BCBM by increasing the expression of NFATc1, RANKL, IL-1β, PTHrP, and other factors. The specific mechanism of miRNA-16's action requires further investigation [100]. miR-378 can directly target BMP-2, inhibit its expression, and induce osteoclastogenesis [101]. Their prognostic value was discovered by Ell et al. who detected four significantly upregulated miRNAs (miR-16, miR-211, miR-378, and Let-7a) during osteoclast formation. Subsequent animal experiments showed that only miR-378 and miR-16 were elevated in mice with high bone metastatic tumor burden. Using real-time fluorescent quantitative RT-PCR to test the serum of 21 healthy women and 38 BCBM patients, they found that miRNA-16 and miRNA-378 were elevated in the bone metastasis group [102].

miRNA-124 has been found to inhibit BCBM by regulating the bone microenvironment. In vitro experiments have shown that miRNA-124 downregulates cytokines that stimulate osteoclastogenesis, inhibits the survival and differentiation of osteoclast precursors, and thus suppresses bone resorption [103]. Furthermore, a previous study has found that miRNA-124 inhibits NFATc1 and regulates the RANKL pathway [104]. Li confirmed its potential for diagnosing breast cancer [105]. Subsequently, the predictive value of miRNA-124 for bone metastasis was discovered by Wei-Luo Cai. They reported that miRNA-124 is downregulated in breast cancer tissue compared to normal tissue and further reduced in bone metastatic tissue. Downregulation of miRNA-124 is associated with invasiveness and shorter bone metastasis-free survival [103].

miRNA-429, an RNA whose high expression is also associated with a good prognosis, plays a role in BCBM microenvironment regulation. In vitro experiments have shown that miRNA-429 downregulates CrkL and MMP-9, two important molecules that promote bone metastasis, and promotes osteoblast differentiation. In animal models, miRNA-429 regulates BCBM microenvironment through similar mechanism [105]. Using ISH and qRT-PCR, Zhang et al. found significantly decreased expression of miRNA-429 in BCBM compared to breast cancer tissue. Moreover, higher levels of miRNA-429 are associated with longer bone metastasis-free survival. Breast cancer patients with low expression of miRNA-429 have a heavier disease burden, manifested by more severe preoperative bone pain and more bone metastatic sites [106].

LncRNA FGF14-AS2 is another RNA that can inhibit breast cancer metastasis [107]. In the context of bone metastasis, FGF14-AS2 inhibits the translation of RUNX2, thereby reducing the transcription of RANKL. Using qRT-PCR, researchers found that FGF14-AS2 is significantly downregulated in breast cancer tissue, and breast cancer patients with low levels of LncRNA FGF14-AS2 have a worse prognosis in terms of distant metastasis-free survival [108] (Table 4).

**Table 4:** Prognostic non-coding RNA biomarkers for BCBM

Biomarker	Increased or decreased	Clinical samples	Potential molecular mechanism	Refs.
miRNA-19a (combined with IBSP)	Increased	23 serum samples from patients with ER-positive breast cancer and 22 samples from healthy individuals	IBSP recruits osteoclast precursor cells and enhances their uptake of exosomes containing miRNA-19a miRNA-19a promotes osteoclast differentiation	[98]
miRNA-30b-5p	Increased	82 samples of primary breast cancer tissue and 93 samples of metastatic breast cancer tissue 20 peripheral blood samples from patients with localized breast cancer and 25 samples from patients with advanced breast cancer	miRNA-30b-5p may be associated with EMT	[99]
miRNA-16,miRNA-378	Increased	21 serum samples from healthy women and 38 serum samples from breast cancer patients with bone metastasis	miRNA-16 and miRNA-378 enhance osteoclast function	[100–102]
miRNA-124	Decreased	79 paired samples of primary breast cancer lesions and non-tumor breast tissue adjacent to the cancer, as well as 34 samples of BCBM	miRNA-124 inhibits the survival and differentiation of osteoclast precursor cells and regulates the RANKL pathway. It also inhibits the proliferation and migration of breast cancer cells	[103–105]
miRNA-429	Decreased	21 bone metastasis samples, including 7 paired samples of bone metastasis and primary breast cancer	miRNA-429 promotes osteoblast differentiation and downregulates the expression of CrkL and MMP-9	[106]
LncRNA FGF14-AS2	Decreased	39 paired samples of breast cancer and non-tumor tissue adjacent to the cancer	LncRNA FGF14-AS2 inhibits breast cancer metastasis and suppresses the expression of RUNX2, thereby inhibiting osteoclast differentiation	[107, 108]

Multiple signatures are not included

It has been reported that a panel of non-coding RNAs may have prognostic potential. eRNAs are short non-coding RNAs transcribed from enhancer sites [109]. Li analyzed RNA-seq data obtained from 1211 primary breast cancer cases and 17 bone metastasis tissues from TCGA (The Cancer Genome Atlas) using Cox regression and LASSO (Least Absolute Shrinkage and Selection Operator) regression. The study explored the relationship between eRNAs and clinical features, immune cell infiltration, and prognosis, and identified seven eRNAs with independent prognostic significance for BRCA bone metastasis: SLIT2, CLEC3B, LBPL1, FRY, RASGEF1B, DST, and ITIH5 [110].

Research of BCBM has identified several genes and mRNAs as biomarkers. Copy number alterations of *MAF* gene at 16q23 mediate bone metastasis in breast cancer. The transcription factor MAF acts on the PTHrP P1 promoter, thereby promoting bone metastasis. Using immunohistochemistry to detect MAF protein levels and FISH to detect 16q23 gains, Pavlovic et al. found that patients with this copy number variation in 334 primary breast cancers had a significant risk of bone metastasis, while no correlation was found with metastasis in other sites [111].

High expression of zinc-finger protein 217 (ZNF217) in MDA-MB-231 breast cancer cells is associated with EMT and certain gene dysregulation [112]. These genes are associated with bone remodeling and osteolytic bone metastasis [113, 114]. In addition, ZNF217 can activate the BMP/Smad signaling pathway upstream, promoting bone metastasis [112, 115]. The role of BMP in cancer metastasis is complex, as it can both repress and promote tumors depending on the context [116]. If drugs targeting ZNF217 are developed, the impact of this dual effect on treatment efficacy should be taken into consideration. Bellanger et al*.* used RT-qPCR to detect *ZNF217* mRNA expression in 113 breast cancer samples and found that high expression of ZNF217 is associated with a higher risk of isolated bone metastasis in ER+ patients, but the level of ZNF217 cannot predict metastasis in other subtypes of breast cancer [112].

Enhancer of zeste homolog 2 (EZH2), as a transcriptional co-repressor of RNA Pol II, increases the expression of integrin β1, leading to focal adhesion kinase (FAK) activation and activation of the TGFβ/Smad2 pathway, promoting bone metastasis [117]. EZH2 has also been found to enhance the stemness and metastatic ability of breast cancer, facilitating metastasis from bone to distant organs [57]. Zhang et al. validated the association between EZH2 expression and bone metastasis-free survival in breast cancer patients using patient information from the GSE dataset. The study found a negative correlation (r = − 0.2394, P = 0.03) between EZH2 expression and bone metastasis, indicating a higher risk of bone metastasis in patients with high EZH2 expression in primary breast cancer [117].

Savci-Heijink et al. identified 15 genes (APOPEC3B, ATL2, BBS1, C6orf61, C6orf167, MMS22L, KCNS1, MFAP3L, NIP7, NUP155, PALM2, PH-4, PGD5, SFT2D2, and STEAP3) associated with the development of bone metastasis in 157 cases of primary breast cancer. This gene signature showed good predictive value for both ER-positive and ER-negative tumors. In a training set and a validation set containing 376 breast tumors, they identified bone metastasis in over 80% of cases. Among these 15 genes, three upregulated genes (NAT1, BBS1, and PH-4) were associated with EMT in tumors, while the other 12 genes were downregulated in bone metastasis [118]. Additionally, PH-4 promotes breast cancer development by altering the extracellular matrix [119]. Recent studies have also shown that NAT1 promotes bone metastasis in luminal-type breast cancer by regulating the bone microenvironment through the NF-κB/IL-1B signaling pathway [27].

Another combination of five genes (HSP90AA1, SPP1, IL3, VEGFA, and PTK2) has been reported to have prognostic significance. ROC analysis demonstrated that this gene signature had a predictive accuracy of over 90% for bone metastasis in breast and lung cancer patients and could be detected using liquid biopsy. Mechanically, HSP90, PTK2, and VEGF regulate cancer cell invasion, proliferation, and metastasis, while IL3 and SPP1 regulate osteogenesis [120].

A study conducted by Cosphiadi et al. included 92 patients with advanced breast cancer, of which 46 patients had bone metastasis. Comparing the 17 patients who only had bone metastasis with non-bone metastasis patients, the researchers identified 17 significantly altered genes. They further confirmed the genes associated with an increased risk of bone metastasis, namely ESR1, PGR, BCL2, REPS2, NAT1, GATA3, ANXA9, C9orf116. The combination of these eight genes had an AUC of 0.928, demonstrating better predictive ability than individual genes [121].

IL-6 gene signature was identified by Mp1nichal Rajski et al. Researchers first investigated the impact of cell–cell interactions on overall gene expression in a co-culture experiment using breast cancer cells and osteoblasts. Co-culturing significantly increased the expression of IL-6. Subsequently, these results were applied to 295 early breast cancer specimens from the Netherlands Cancer Institute to evaluate the clinical relevance of the in vitro experiment. The researchers confirmed an IL-6 gene signature composed of 72 genes, which was associated with shorter time to bone metastasis in patients with high expression of this gene signature [122].

A study analyzed the expression of TFF1 and verified its positive correlation with tumor relapse to bone using SAM analysis and quantitative RT-PCR. They used PAM analysis to identify a 31-gene signature predicting bone metastasis with 100% sensitivity and 50% specificity [123].

By comparing gene expression between different sublines of human breast cancer cell line MDA-MB-122, researchers identified four highly overexpressed genes in the bone metastasis group: IL11, CTGF, CXCR4, and MMP-1. They further demonstrated that the expression of these factors was further enhanced by the pro-metastatic cytokine TGFβ, providing evidence for these genes as potential diagnostic markers and therapeutic targets [114].

Woelfle et al. reported significant differences in gene expression between bone metastasis-positive and -negative patients' primary tumors based on cDNA microarray expression analysis. The differentially expressed genes were involved in extracellular matrix remodeling, adhesion, and cytoskeletal plasticity. They then identified a 73-gene signature capable of identifying lymph node-negative patients with or without bone marrow micrometastases. Immunohistochemical analysis of a test set containing 83 primary breast tumor samples, from patients with or without tumor cells in the bone marrow, confirmed that patients with decreased expression of CK8, CK18, or CK19 had an increased incidence of bone marrow micrometastases [124].

Ray investigated the prognostic value of FOXC1 gene signature in basal-like breast cancer metastasis. They found that ectopic overexpression of FOXC1 increased breast cancer cell proliferation, migration, and invasion. They constructed a FOXC1 gene signature composed of 30 genes that could predict overall survival and brain metastasis occurrence in patients. Subsequent analysis of a dataset containing 286 samples explored the relationship between *FOXC1* mRNA expression and metastasis. Higher FOXC1 expression was positively correlated with brain metastasis (P = 0.02) but negatively correlated with bone metastasis (P = 0.0002) [125].

The tumor suppressor gene Raf kinase inhibitory protein (RKIP) inhibits downstream genes involved in BCBM (MMP1, OPN, CXCR4) through miRNA let-7. Using an independent dataset of breast cancer patients, researchers found that patients with high expression of downstream RKIP pathway metastasis genes and low expression of RKIP had a significantly increased risk of metastasis [126].

The function of BMP has been discussed previously. It is also found to compose a prognostic gene signature along with other genes. Research based on the construction of a weighted co-expression network of differentially expressed genes (DEGs) related to EMT in metastatic breast cancer identified hub genes such as FERMT2, ITGA5, ITGB1, MCAM, CEMIP, HGF, TGFBR1, and F2RL. Differential expression of BMP2, BMPR2, and GREM1 was found in two datasets of BCBM. Based on the results of bioinformatics analysis from this study, it is speculated that BMP-2 may regulate immune infiltration processes in breast cancer tissue through the PI3K/Akt signaling pathway, thereby affecting cancer prognosis [127].

As has been discussed earlier, breast cancer cells secrete MSP, which acts on the RON receptor and promotes osteoclast activation [46]. Welm et al. team used a mouse model of breast cancer to demonstrate that high expression of MSP promotes osteolytic metastasis of cancer cells from the primary tumor site to the bone. Subsequently, using microarray gene expression data, they found that tumors with high expression of MSP/MT-SP1/MST1R had a significantly increased incidence of bone metastasis, and demonstrated that the overexpression of MSP pathway genes has independent prognostic value for breast cancer patients in terms of metastasis and death [128].

Using microarray datasets from GSE2034, GSE2603, GSE12276, and NKI295, Li constructed a Gene Dependency Network and selected 51 genes associated with breast cancer metastasis. Survival analysis using training and testing sets showed that the identified gene signature was associated with shorter bone metastasis-free survival in high-risk cancer patients, confirming the prognostic value of this gene panel [129].

Fan used GSE124647 as the training set and GSE16446, GSE45255, GSE14020 as the validation sets to explore the differential expression levels of key genes between breast cancer non-bone metastasis and bone metastasis groups. Potential prognostic-related genes were analyzed, validated, and a columnar line graph (GESBN) model based on gene expression features was constructed to predict the likelihood of bone metastasis in breast cancer patients. Patients were sorted based on risk scores from the training set and divided into high-risk and low-risk groups for bone metastasis. The GESBN model constructed using GAJ1, SLC24A3, ITGBL1, and SLC44A1 indicated a poorer overall survival (OS) for high-risk individuals. The GESBN model constructed using GJA1, IGFBP6, MDFI, TGFBI, ANXA2, and SLC24A3 indicated a poorer PFS for high-risk individuals. Two hub genes, SLC44A1 and MDFIBC, were also selected and may serve as therapeutic targets for BCBM [130].

Although these gene signatures cover many genes, the overlap between them is relatively small. Possible reasons for this phenomenon include different sample types (breast cancer cell lines or tissues), choice of experimental methods, and selection of patient types [131]. However, this phenomenon may also be related to the heterogeneity of breast cancer. On the one hand, different spatial locations within the same tumor may contain subpopulations of breast cancer cells with different molecular characteristics [132, 133]. On the other hand, BCBM involves multiple stages of development, with corresponding genes involved at different stages [134]. Therefore, more precise study of the spatiotemporal evolution of breast cancer in bone metastasis may help address this issue.

#### Circulating cells

Circulating tumor cells (CTCs) are tumor cells that detach from the tumor and circulate in the bloodstream. Circulating tumor cells can be collected and used for liquid biopsy assays [135]. In patients' blood samples, CTCs are identified by using immunomagnetic-based assays to isolate cells that lack CD45 and are positive for cytokeratin and epithelial cell adhesion molecule (EpCAM). Patients who test positive for CTCs at both pre-chemotherapy and post-chemotherapy time points have a higher proportion of bone metastasis compared to patients who are CTC negative at both time points (21.0% vs. 37.5%) [136]. Similar methods have shown a significant increase in CTC numbers in patients with bone metastasis, which is independent of bone metastasis occurrence [137]. Elnagdy et al. examined the RNA expression of CTCs in breast cancer patients and found a correlation between high expression of *TFF1* mRNA and bone metastasis. Thus, the specific gene expression of CTCs can be used in bone metastasis prediction [138]. Furthermore, it has been reported that CTCs from breast cancer patients with bone metastasis exhibit active androgen receptor (AR) signaling pathway. Therefore, isolating CTCs from peripheral blood of breast cancer patients and detecting AR expression may be used to predict the occurrence of bone metastasis [139]. These studies suggest that the information carried by CTCs may indicate the bone tropism of tumors or the risk of bone metastasis in patients.

Osteocalcin is a marker of late osteoblast differentiation. Circulating osteocalcin-positive cells (cOC) are small mononuclear cells expressing osteocalcin in peripheral blood mononuclear cells. Researchers have identified the optimal cutoff value for cOC as 0.069% based on the ROC curve. Patients with high cOC levels have significantly shorter bone metastasis-free survival compared to those with low cOC levels, indicating that cOC can predict early bone metastasis. However, cOC levels do not increase in advanced bone metastasis patients. Animal models have also shown a significant increase in cOC during early bone metastasis, which gradually decreases with tumor progression. Researchers have also found that benign fractures do not affect the elevation of cOC related to metastasis, which is an advantage of using cOC as a predictor of bone metastasis [140].

In addition to the mentioned biomarkers, there are other factors that may have prognostic significance. Tavazoie et al. compared primary tumors resected from 11 patients with lung, bone, or brain metastases to tumors resected from 9 patients without metastatic recurrence. They found that patients with low expression of miRNA-335 and miRNA-126 in the primary tumor were more likely to develop metastasis [141]. Additionally, low expression of miRNA-30a, miRNA-30d, and miRNA-30e was associated with poor recurrence-free survival [142]. Therefore, miRNA-335, miRNA-126, and members of the miRNA-30 families could also serve as markers for BCBM. In a study using bioinformatics methods, key genes and long non-coding RNAs related to BCBM were identified. They constructed a differentially expressed lncRNA–mRNA interaction network and analyzed node degrees to identify core driver genes. They suggested that RP11-317-J19.1, PTP4A1, and BNIP3 may play key roles in suppressing cancer invasiveness and serve as strong predictors of BCBM [143]. It has been reported that osterix (Osx) upregulation is associated with lymph node metastasis and negatively correlates with overall survival. Knocking down Osx suppresses breast cancer invasion and osteolytic metastasis by downregulating MMP9, MMP13, VEGF, IL-8, and PTHrP, indicating the involvement of Osx in breast cancer invasion, angiogenesis, and bone resorption. This study demonstrates the potential value of Osx in predicting lymph node metastasis in breast cancer [144]. Considering its role in bone metastasis, Osx may be worth investigating as a predictor of BCBM in clinical samples. Retinoic acid-induced 2 (RAI2) is a protein involved in maintaining the differentiation state of ERα-positive breast cancer cells, and Werner et al*.* found that downregulation of RAI2 is associated with early bone metastasis in ERα-positive breast tumors, suggesting its potential role in inhibiting early hematogenous dissemination of tumor cells to the bone marrow [145]. Subsequent studies have also demonstrated the prognostic value of RAI2 in breast cancer [146]. Whether RAI2 can predict bone metastasis in breast cancer requires further validation in clinical samples.

### Predictive biomarkers

Predictive biomarkers can predict a patient's response to treatment or be used for monitoring during treatment to determine if the patient is benefiting from the therapy [75].

#### Proteins

CAPG and GIPC1 have been confirmed to have prognostic significance by Westbrook et al. In the same study, they also demonstrated the predictive role of these two molecules. Patients with high expression of CAPG and GIPC1 treated with zoledronic acid showed a tenfold reduction in the risk of bone metastasis compared to the control group (P = 0.008). Therefore, detecting CAPG and GIPC1 can help identify patients who would benefit from zoledronic acid treatment [83].

In a previously mentioned AZURE clinical trial, high levels of DOCK4 were found to be associated with bone metastasis in the control group but not statistically correlated in the group treated with zoledronic acid. This indicates that the use of zoledronic acid can effectively reduce the risk of bone metastasis in patients with high expression of DOCK4 [84].

Urinary N-terminal telopeptide (uNTX) is a marker reflecting bone resorption [95]. A retrospective analysis conducted by Allan Lipton used ELISA to detect NTX in the urine of 1705 breast cancer patients. The results suggested that patients with uNTX levels above the median after 3 months of denosumab or zoledronic acid treatment had a higher risk of reduced overall survival and increased risk of disease progression [147]. Another prospective cohort study further elucidated the predictive value of uNTX. NTX in the urine of patients (71 breast cancer patients with bone metastasis, including 39 with extraskeletal metastasis) was detected using a similar method. After 3 months of zoledronic acid treatment, high levels of NTX (NTX > 100 nmol BCE/mmol creatinine) in patients with bone metastasis only indicated poor prognosis, consistent with Allan Lipton's conclusion. The researchers also found a strong correlation between NTX levels at 1 month and 3 months after zoledronic acid treatment and long-term NTX levels at 12 months. Therefore, the prognosis of these patients can be identified early after initiating zoledronic acid treatment. However, the predictive ability of NTX can be affected by metastases in other sites, as patients with extraskeletal metastasis show unstable NTX levels that are not associated with survival [148].

#### Genes

The prognostic value of MAF has been discussed earlier. Subsequent studies have found that MAF may be used to guide treatment for BCBM. Using FISH to detect MAF in primary tumor samples, Robert Coleman defined copy number >  = 2.5 as MAF+ve and studied a cohort of 1769 patients, including those from the AZURE trial. Kaplan–Meier survival curve analysis showed that MAF-ve tumor patients benefited from zoledronic acid, while in premenopausal women with MAF+ve tumors, zoledronic acid treatment led to poorer overall survival (OS) [149]. This result suggests a correlation between menopausal status in women and BCBM, which may be related to hormonal changes as studies have shown that estrogen induces apoptosis in osteoclasts, and other hormones involved in perimenopausal changes regulate the bone microenvironment [150, 151]. Predictive power of MAF might be further improved when we take other factors into consideration, for example, IL-6 expression. Studies have shown that IL-6, a factor involved in BCBM, significantly increases in the serum of women with age. There is evidence that IL-6 may be mechanistically related to the function of zoledronic acid. Zoledronic acid alters the expression of IL-6 in prostate cancer cells and influences osteoclast differentiation through IL-6 [152, 153]. Therefore, it can be speculated that IL-6 may be one of the factors contributing to the differential efficacy of zoledronic acid among patients with different menopausal statuses. Further research and validation is required to determine how MAF, menopausal status, and IL-6 can be used in conjunction to inform treatment decisions. This represents one of many similar avenues of study that can help bring biomarkers from the bench to the bedside.

**Fig. 7:**
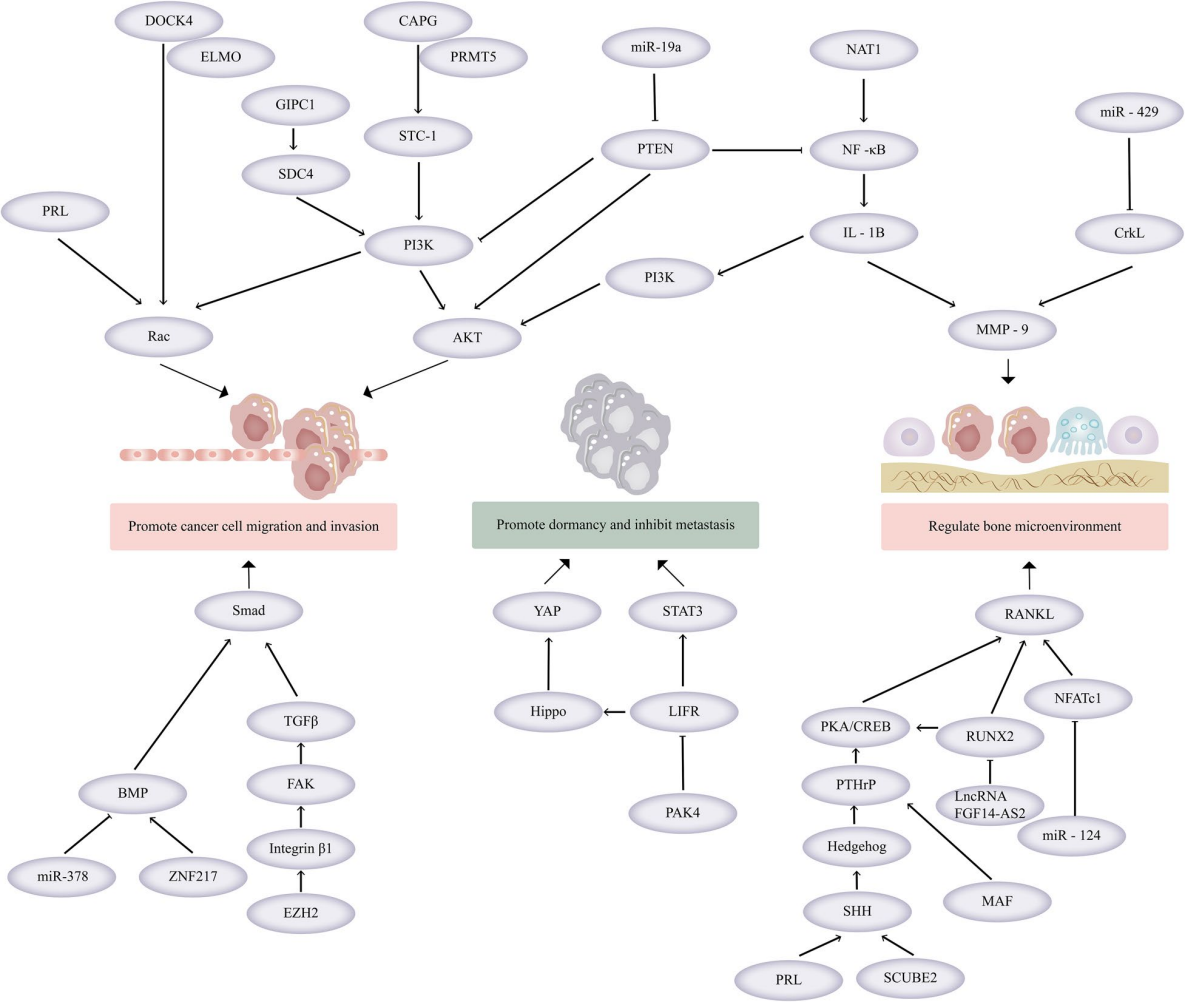
Relevant signaling pathways of biomarkers in BCBM. Multiple signatures are not included

These biomarkers not only have predictive and prognostic value but also participate in the pathophysiology of BCBM (Table 5). Their related signaling pathways are shown in Fig. 7.

**Table 5:** Biomarkers and their functions

Functions	Biomarkers	Refs.
Cell adhesion and cytoskeleton	GIPC1, DOCK4, CAPG, IL-1B, PRL	[79, 82, 85, 91, 94]
Tumor stemness or EMT	PAK4, RANKL, IL-1B, EZH2, ZNF217	[57, 78, 87, 93, 112]
Regulation of the bone microenvironment	RANKL, PRL, ZNF217, miRNA-19a, IBSP, miRNA-124, miRNA-429, LncRNA FGF14-AS2, MAF, miRNA-16, miRNA-378, SCUBE2	[28, 86, 89, 98, 100, 101, 103, 104, 106, 108, 111, 112]
Bone turnover markers	P1NP, CTX, 1-CTP, NTX	[96, 147]

Multiple signatures are not included

A subset of the aforementioned biomarkers have been evaluated for use in liquid biopsies. The technique of liquid biopsy involves real-time combined analysis of circulating tumor cells, circulating tumor DNA, extracellular vesicles, RNA, proteins, and other substances derived from primary or metastatic tumor lesions in blood or other body fluids, providing information about the tumor [154]. It offers a variety of advantages over tissue biopsy.

Within the scope of liquid biopsy, the development of novel assays to detect biomarkers within exomes is particularly promising. Exosomes are a subset of extracellular vesicles that mediate cell-to-cell communication by transporting molecular cargoes such as proteins, mRNA, miRNA, and lipids from donor cells to recipient cells, thereby participating in physiological and pathological processes. Exosomes offer several advantages in liquid biopsy. First, they are enriched in body fluids and easily accessible. Second, due to their lipid bilayer structure, exosomes are stable, which allows them to maintain stability during circulation in the body, sample transportation, and storage, making them crucial for the development of biomarkers using retrospective samples [155, 156].

Numerous studies have enriched and purified exosomes through ultracentrifugation and confirmed their role in the process of BCBM and their potential as biomarkers. As mentioned earlier, exosomal miRNA-21 promotes the formation of osteolytic metastatic lesions in breast cancer, and exosomal mRNA of miRNA-19a, HSP90AA1, SPP1, IL3, VEGFA, and PTK2 genes have also been validated as biomarkers [20, 98, 120]. In addition to exosomes, free RNA, proteins, and circulating cells can also be used for liquid biopsy in the prognosis of BCBM, and they are summarized in Table 6. A recent study has reported that serum periostin is a prognostic biomarker for breast cancer-specific survival [157]. Previous research has also indicated high expression of periostin in breast cancer patients with bone metastasis [158]. Therefore, future studies can validate whether periostin can be applied in liquid biopsy to predict the risk of bone metastasis occurrence in high-risk individuals [157].

**Table 6:** Potential biomarkers for liquid biopsy in breast cancer bone metastases

Biomarkers	Category	Techniques used	Refs.
RANKL	Protein	ELISA	[88]
SCUBE2	Protein	ELISA	[28]
P1NP, CTX, 1-CTP	Protein	Immunological analysis	[96]
uNTx	Protein	ELISA	[147]
IBSP Exosomal miRNA-19a	RNA, protein	ELISA (IBSP) RT-qPCR (Exosomal miRNA-19a)	[98]
miRNA-30b-5p	RNA	RT-qPCR	[99]
miR-378 and miR-16	RNA	RT-qPCR	[102]
HSP90AA1, SPP1, IL3, VEGFA, PTK2	Gene panel	RT-qPCR	[120]
CTC	Cell	Immunomagnetic sorting	[136, 137]
cOC	Cell	Flow cytometry and immunofluorescence staining	[140]

Liquid biopsy offers unique advantages compared to tissue biopsy. Tumor cells are heterogeneous, and cancer cells undergo molecular changes because of fluctuations in microenvironment and therapeutic interventions. Whereas tissue biopsies are limited in their ability to be representative of subpopulations of tumor cells and to reflect real-time development of the tumor, liquid biopsies are not [156]. The major benefit of liquid biopsy is that it is minimally invasive and can be performed multiple times [159]. Therefore, the use of liquid biopsy helps in closely monitoring patients, predicting the risk of BCBM, and guiding treatment decisions.

## Single-cell sequencing and organoid models in the field of breast cancer bone metastasis (BCBM) research

### Single-cell sequencing

Technological advancements are making nucleic acids increasingly relevant as the targets of screening and diagnosis. A multistage disease as complex and heterogenous as BCBM offers a particular set of challenges that must be addressed by a deliberate, well planned sequencing protocol. The continuous evolution of heterogeneous tumor cell populations resulting in the emergence of treatment-resistant clones, which can cause drug responses in the primary site and the distant metastatic sites to be very different [160]. Traditional high-throughput bulk sequencing techniques provide only average transcriptomic levels of all cells in heterogeneous tumor tissues and is of relatively low resolution. However, single-cell transcriptomics offers high resolution and overcomes the limitations of traditional bulk sequencing techniques, allowing for the study of gene expression states and functions at the single-cell level. Single-cell sequencing can classify breast cancer cell populations of different molecular subtypes to identify disease patterns that may be associated with adverse prognosis and drug resistance. This technology can also be used to elucidate tumor microenvironment heterogeneity, laying the foundation for further understanding the origin, metastasis, and treatment resistance of tumors. Single-cell sequencing reveals tumor heterogeneity and evolution, promoting the development of precision medicine [161, 162].

Wu et al*.* conducted a study employing single-cell RNA sequencing to delve into the cellular components within the microenvironment. Their research uncovered that SCUBE2, expressed by MCF7 cells, plays a pivotal role in inducing the enrichment of a specific osteoblastic subpopulation within metastatic lesions. This induction, in turn, promotes osteoblast differentiation [28]. In a separate investigation utilizing data gathered through single-cell sequencing, Zhang et al*.* explored various cell subpopulations within the tumor microenvironment of bone metastasis. Their research pinpointed the existence of the MSC_MARCKSL1 subpopulation, situated in the early stages of differentiation and characterized by its significant differentiation potential. These findings suggest a potential involvement of this subpopulation in the regulation of bone metastasis [163].

Sequencing techniques can also provide important insight into how breast to bone metastasis influences further metastasis to other sites [5]. The origin of metastasis to other organs can be from the primary tumor or from bone metastatic lesions, highlighting the need to understand the tumor's evolutionary process. Brown et al. utilized phylogenetic techniques to analyze whole-exome sequencing and copy number variation data in breast cancer specimens, exploring the patterns of tumor metastasis. Their study observed that the most common pattern of initial metastasis in breast cancer is a single metastatic site, followed by multiple seeding events from the primary tumor, which subsequently undergo metastasis-to-metastasis cascading dissemination [164].

Single-cell sequencing plays a pivotal role in advancing the development of biomarkers, particularly in breast cancer research. In pertinent studies, breast cancer cells and immune cell populations of distinct molecular subtypes have been systematically classified, leading to the identification of unique subgroups that may hold relevance for prognosis and the understanding of drug resistance [162]. For instance, Savas et al*.* conducted single-cell RNA sequencing on T cells isolated from human breast cancer tissues. This endeavor revealed the remarkable heterogeneity within the T cell population and culminated in the development of a CD8^+^ TRM gene signature, derived from the sequencing data. This signature serves as a valuable prognostic biomarker, specifically tailored for early TNBC patients [165]. Moreover, investigations into breast cancer tumor stem cells using single-cell sequencing have unveiled a set of 14 genes that exhibit significant upregulation in tumor stem cells. Notably, these genes have been correlated with survival outcomes, positioning them as potential biomarkers with clinical significance [166].

Single-cell sequencing not only holds promise for predicting patient prognosis but also plays a vital role in predicting drug efficacy. An illustrative example of this is the work of Wang et al*.*, who utilized this technology to uncover the presence of immune-inhibitory immature myeloid cells (IMCs) within breast cancer tumors that displayed resistance to anti-Her2 treatment and the CDK4/6 inhibitor Palbociclib. To surmount this resistance, Wang's research demonstrated the effectiveness of a combined approach. It involved the use of tyrosine kinase inhibitors that targeted IMCs in conjunction with immune checkpoint inhibitors. This innovative combination therapy successfully overcame resistance, highlighting the considerable potential of single-cell sequencing in monitoring the evolution of tumors under the influence of drug interventions. It offers valuable insights into devising more effective treatment strategies [167].

Knowledge of the mechanisms behind BCBM is only as good as the in vitro and in vivo experimental models used. Development of more suitable BCBM models is critical to both the development of better screening biomarkers and therapeutic drugs. One of the most promising avenues in the study of BCBM is the use of organoids in precision medicine. Organoids are three-dimensional models containing multiple cell types that simulate the characteristics of tumors in the body and can be used to study the biological properties of tumor cells in detail [168]. They offer unique advantages since they are often cheaper and faster to use than in-vivo models. Previous studies have found that the use of xenograft organoid models can be used for drug screening and precision therapy. In a case study of an early-stage metastatic TNBC patient, researchers observed that the model's response to drugs correlated with the clinical outcomes observed [169]. The development of more sophisticated organoids may yield a better temporal understanding of BCBM since sample sizes can be larger, and researchers can afford to do perform histological analysis at smaller time intervals. In a recent study using organoid models derived from BCBM patients, single-cell RNA sequencing (ScRNAseq) results showed that the model largely preserved cellular subclonal heterogeneity. These findings demonstrate that organoid models may be uniquely valuable in studying tumor heterogeneity without sacrificing authenticity [161].

### Organoids models

Organoids represent intricate three-dimensional structures that closely mimic the architecture and functionality of organs. They originate from stem cells or organ-specific progenitor cells and are generated through in vitro self-organization processes. When compared to other tumor models, organoids offer conditions that more faithfully replicate the characteristics of the original tumor. This is especially advantageous in the in-depth study of human cancers, including breast cancer. To illustrate, when cultured in conventional tissue culture bottles, there exists a substantial disparity, exceeding 17 million times, between the stiffness of healthy mammary glands and the surrounding cell layer. In contrast, the elastic modulus of Matrigel, a common substance used in organoid culture, is relatively like that of mammary glands. This aligns organoid cultures more closely with the natural tissue environment. Furthermore, in standard two-dimensional (2D) cell cultures, there is a gradual loss of heterogeneity, whereas the tumor environment maintains a high degree of heterogeneity. In contrast, organoids encompass various cell types and can simulate some of the essential functions of organs. As a result, they are exceptionally well-suited for research into the tumor microenvironment (TME) and offer a more comprehensive and physiologically relevant platform for such investigations [170].

The extracellular matrix (ECM) plays critical roles in driving cancer phenotype, disease progression and therapeutic response in vivo. 3D engineered matrices with tunable biochemical and mechanical properties are poised to answer previously untestable hypotheses surrounding mechanisms of these important cancer–matrix interactions [171]. Dhimolea et al. employed organoids as a valuable tool to investigate hormone therapy resistance within the intricate metastasis microenvironment, a context that is challenging to replicate in traditional 2D cultures. In their study, they cultured spheroids derived from hormone receptor-positive (HR+) breast cancer and prostate cancer cells, as well as patient-derived organoids, within a 3D ECM. They examined these cultures both in isolation and in conjunction with bone marrow matrix. Through this approach, they made a significant discovery. Their research revealed the extent to which tumor cells rely on hormone receptor (HR) signaling for anchorage-independent growth. Furthermore, it underscored how the metastatic microenvironment has the capacity to restore this malignant property of cancer cells, particularly during hormone therapy. This finding sheds valuable light on the mechanisms underlying hormone therapy resistance and provides insights into the dynamic interplay between cancer cells and their microenvironment in the context of metastasis [172].

Organoids serve as invaluable tools that offer histological insights into cancer development, enhance our understanding of the carcinogenic process, and open avenues for the discovery of novel treatment approaches. Notably, organoid models have found application in the investigation of bone metastasis in breast cancer. In some studies, researchers have identified a correlation between elevated expression of Sclerostin (SOST) and the occurrence of bone metastasis in breast cancer, as well as a poorer prognosis for breast cancer patients. Silencing SOST expression has been shown to significantly diminish the capacity of SCP2 cells. Moreover, it has been revealed that the interaction between SOST and STAT3 can potentiate the TGF-β/KRAS signaling pathway, consequently promoting tumor growth and bone metastasis. Remarkably, the administration of S6, a leading candidate drug, has demonstrated a notable capacity to inhibit the growth of breast cancer organoids. Additionally, it has exhibited efficacy in mitigating bone metastasis in mouse models. These findings underscore the potential therapeutic value of targeting SOST and its associated pathways in addressing breast cancer and its metastatic spread to the bone [173]. Recent research has underscored the pivotal role of Wnt ligand signaling in the regulation of migration, invasion, and metastasis in breast cancer. Specifically, the Wnt receptor frizzled 6 (FZD6) has emerged as a valuable marker for identifying TNBC patients who face a heightened risk of metastasis and recurrence. In this study, organoids were leveraged as a crucial tool to assess the transformation degree of breast cancer cells. This approach allows for a deeper understanding of the molecular and functional alterations that occur in breast cancer, shedding light on the potential mechanisms underlying metastasis and offering valuable insights into disease progression and patient risk assessment [174].

While organoids hold great promise as invaluable models, their practical implementation faces certain limitations. Firstly, despite their ability to offer more realistic 3D models for cancer research, creating an organoid model that faithfully mirrors the heterogeneity of a patient's tumor and its tumor microenvironment (TME) remains a significant challenge. This challenge arises due to the absence of critical components such as stroma, immune cells, blood vessels, and microbiota within organoids. Additionally, the time required for culturing organoids derived from breast cancer patients poses a significant hurdle. The process can be time-consuming, and the efficiency of organoid derivation and reliable in vitro expansion is often characterized by unpredictability and low success rates. These limitations underscore the need for continued research and development efforts to refine organoid models and enhance their capacity to faithfully recapitulate the complexity of tumors and their microenvironments [170, 171].

## Conclusions and future perspectives

Bone metastasis is a serious complication that occurs in advanced breast cancer, impairing patients' quality of life and reducing their lifespan. Existing detection methods often struggle to identify early-stage bone metastasis, and many patients are only diagnosed when symptoms arise [175].

In this context, many studies have attempted to explore biomarkers that can predict the occurrence of bone metastasis, enabling the selection of high-risk patients for subsequent follow-up and treatment. Among them, biomarkers compatible with liquid biopsy offer unique advantages over tissue biopsy since measurements can be repeated and assess the risk of bone metastasis multiple times. Future research endeavors in the realm of liquid biopsy are poised to unlock the full potential of these biomarkers for longitudinal monitoring during disease progression and therapeutic interventions. Such studies hold significant promise, particularly in the context of assessing the risk of bone metastasis in breast cancer patients, enabling precise patient stratification, and facilitating informed drug selection. An illustrative example of the potential of longitudinal liquid biopsy comes from the retrospective study conducted by Chin et al., involving 33 breast cancer patients. In this study, serial analysis of circulating tumor DNA (ctDNA) was employed to monitor the status of bone metastatic lesions and to detect acquired genetic alterations linked to drug resistance. The outcomes of this investigation highlight the clinical feasibility and utility of serial ctDNA analysis in tracking tumor responses to CDK4/6 inhibitors. Consequently, this study provides compelling evidence supporting the practical application of longitudinal liquid biopsy in clinical settings [176]. These findings underscore the immense promise of liquid biopsy as a dynamic tool for monitoring disease progression and treatment response, thereby contributing to more personalized and effective care for breast cancer patients.

Our understanding of the predicting power of the biomarkers discussed is currently limited. Some of the biomarkers mentioned are just beginning to show promise in preclinical testing, whereas others have already demonstrated some degree of utility. Without the use of scaled up clinical trials in large patient populations, it is exceedingly difficult, if not impossible, to determine which biomarkers will be robust enough to predict BCBM. More substantial validation studies are in our future. With continued research in particular patient populations, our understanding of which biomarkers will be clinically useful in which situations will improve.

Concurrently, sequencing techniques will continue to become more sophisticated and will make the goal of precision medicine more attainable. Research in the basic sciences and improved models will also undoubtedly advance the discovery of novel biomarkers and therapeutic compounds while providing valuable insight into how to best use them.

Despite the significant strides made in cancer research and biomarker development facilitated by single-cell sequencing, there are challenges that need to be addressed before the widespread adoption of this promising technology. One such challenge is the limitation imposed by the sheer number of cells within a solid tumor tissue, which often surpasses the capacity for sequencing offered by single-cell sequencing. Additionally, the high costs associated with this technology pose a barrier to its widespread application [177, 178]. Future research efforts may be directed towards enhancing the precision of single-cell sequencing while elucidating the characteristics of the tumor microenvironment and its cellular composition. An avenue to address this challenge lies in the integration of single-cell sequencing with bulk sequencing data. A notable example is the work of Newman et al*.*, who developed CIBERSORT, a tool that employs machine learning and deconvolution algorithms to estimate the composition and abundance of immune cells within mixed cell populations. Building upon this advancement, Guo et al*.* successfully integrated results from both single-cell sequencing and bulk sequencing. This integration enabled them to delve into T-cell heterogeneity in TNBC and construct a prognostic risk model specifically related to T-cells in TNBC. The integration of these data sources holds significant promise for gaining a comprehensive understanding of tumor biology and the immune microenvironment. This approach has the potential to advance the development of precise prognostic models and personalized therapeutic strategies in breast cancer research and beyond [179]. This underscores the necessity of developing corresponding algorithms to efficiently manage the vast amount of data generated by single-cell sequencing.

Another noteworthy limitation is the loss of spatial information [178]. However, this limitation can be addressed through the integration of spatial transcriptomics, which enables the visualization of transcriptomes and quantitative analysis at a single-cell level with spatial resolution. This integration offers a more precise understanding of breast cancer metastasis. In a compelling example, Liu et al. employed multimodal intersection analysis to combine these two technologies. Their work led to the identification of early metastatic subpopulations of breast cancer and their spatial distribution characteristics. Notably, they revealed that this subpopulation exhibited an initial increase followed by a decrease in oxidative phosphorylation pathway activity during the metastasis process, while the glycolytic pathway displayed the opposite trend. These findings underscore the potential significance of cell metabolism in predicting breast cancer metastasis [180].

The added benefit of discovering and developing novel biomarkers is that most of them also play a role in cancer development and can be potential targets for treatment. For example, RANKL has significance as a biomarker and is also the target of denosumab, an approved for the treatment of bone metastasis [86]. Other biomarkers may not currently have known clinical significance but may become appealing targets for BCBM treatment as our knowledge of the molecular mechanisms behind BCBM deepen. IL-1B, another biomarker that was discussed, is showing potential in preclinical models as a treatment target: when used alone the IL-1 receptor antagonist anakinra inhibits bone metastasis, although it also promotes primary tumor growth. The combination of anakinra with doxorubicin and zoledronic acid compensates for this drawback by reducing primary tumor growth while inhibiting bone metastasis [181]. Further research into other biomarkers may reveal even more promising therapeutic targets.

Tremendous strides have been made in unraveling the intricate mechanisms underlying breast cancer bone metastasis. Yet, certain processes, especially those related to secondary metastasis following initial bone metastasis, still lack comprehensive characterization. While existing research has shed light on some aspects, such as the role of the bone metastatic microenvironment in promoting secondary metastasis, there remains a need for deeper understanding. Current studies have pointed to specific mechanisms, such as cancer cell-autonomous secretion of EZH2 and immune suppression mediated by osteoclast-derived apoptotic bodies, both of which can be promising targets for therapy [57, 58]. These findings underscore the potential for novel therapeutic strategies in breast cancer bone metastasis. To advance our knowledge in this field, further exploration of the interactions between various cell subpopulations within the bone microenvironment is essential. This will enable a more comprehensive understanding of the cascade of events that drive breast cancer bone metastasis. Moreover, it will lay the foundation for the development of innovative therapeutic interventions aimed at interrupting these complex processes and improving patient outcomes.

In conclusion, the future trajectory of research in breast cancer bone metastasis should be guided by several key objectives: 1) Mechanistic Understanding: Continued efforts should be dedicated to deepening our understanding of the intricate mechanisms that drive breast cancer bone metastasis. This knowledge will pave the way for more targeted and effective treatments. 2) Biomarker Development: The development of highly accurate and reliable biomarkers is crucial. These biomarkers have the potential to aid in early diagnosis, risk assessment, and treatment stratification for breast cancer patients with bone metastasis. 3) Clinical Validation: It is imperative to subject these biomarkers to extensive large-scale clinical validation to ensure their robustness and reliability. This step is essential before their potential implementation in routine clinical practice. 4) Therapeutic Targets: Exploring whether these biomarkers can also serve as therapeutic targets is a promising avenue of investigation. Targeted therapies based on these biomarkers may offer more effective and personalized treatment options.

By pursuing these objectives, the development of new technologies and research models will undoubtedly advance the study of novel biomarkers, with the potential for clinical application in the near future, ultimately assisting in the prevention of BCBM and improving the prognosis of patients with BCBM.

## Data Availability

Data sharing is not applicable to this article as no datasets were generated or analyzed during the current study.

## Abbreviations

SREs: Skeletal-related events
BCBM: Breast cancer bone metastasis
RANKL: Receptor activator of NF-κB ligand
TNBC: Triple-negative breast cancer
ECT: Emission computed tomography
CT: Computed tomography
MRI: Magnetic resonance imaging
PET-CT: Positron emission tomography-computed tomography
SRS: Src-responsive signature
DKK1: Dickkopf-1
NAT1: *N*-Acetyltransferase 1
SCUBE2: Signal peptide, cubulin domain, epidermal-growth-factor-like protein 2
CXCL12: Chemokine C-X-C motif ligand 12
IGF1: Type 1 insulin-like growth factor
CXCR4: C-X-C chemokine receptor type 4
HSCs: Hematopoietic stem cells
TSP-1: Thrombospondin-1
PTHrP: Parathyroid hormone-related protein
MSP: Macrophage-stimulating protein
OPG: Osteoprotegerin
PFS: Progression-free survival
nPAK4: Nuclear p21-activated kinase 4
LIFR: Leukemia inhibitory factor receptor
YAP: Yes-associated protein
EMT: Epithelial-mesenchymal transition
NMBC: Non-bone metastatic breast cancer
BMBC: Bone metastatic breast cancer
CAPG: Macrophage-capping protein
GIPC1: PDZ domain–containing protein
PRMT5: Protein arginase methyltransferase 5
STC-1: Stanniocalcin-l
SDC4: Syndecan-4
DOCK4: Dedicator of cytokinesis protein 4
ELMO: Engulfment and cell motility
PRL: Prolactin
SHH: Sonic Hedgehog
PRLR: Prolactin receptor
P1NP: Procollagen type I N-terminal propeptide
CTX: C-telopeptide of type-1 collagen
1-CTP: Cross-linked carboxyterminal telopeptide of type I collagen
IBSP: Integrin binding sialoprotein
ZNF217: Zinc-finger protein 217
EZH2: Enhancer of zeste homolog 2
FAK: Focal adhesion kinase
RKIP: Raf kinase inhibitory protein
DEGs: Differentially expressed genes
OS: Overall survival
CTC: Circulating tumor cell
EpCAM: Epithelial cell adhesion molecule
AR: Androgen receptor
cOC: Circulating osteocalcin-positive cells
Osx: Osterix
RAI2: Retinoic acid-induced 2
uNTX: Urinary N-terminal telopeptide
